# FINDRISC modified for Cuba as a tool for the detection of prediabetes and undiagnosed diabetes in cuban population

**DOI:** 10.17843/rpmesp.2024.414.14138

**Published:** 2024-12-03

**Authors:** Eduardo Cabrera-Rode, Oscar Díaz-Díaz, Neraldo Orlandi González, Mohan Ronald

**Affiliations:** 1 Institute of Endocrinology, Universidad de Ciencias Médicas de La Habana, Havana, Cuba. Universidad de Ciencias Médicas de La Habana Institute of Endocrinology Universidad de Ciencias Médicas de La Habana La Habana Cuba

**Keywords:** FINDRISC, LA-FINDRISC, Prediction, Screening, Type 2 Diabetes Mellitus, Prediabetes, Dysglycaemia

## Abstract

**Objectives.:**

To evaluate the Finnish Diabetes Risk Score (FINDRISC) modified for Cuba as a tool for the detection of prediabetes and undiagnosed diabetes in Cuban population.

**Materials and methods.:**

An analytical cross-sectional and secondary source epidemiological study was conducted in 3737 adults aged 19 years and older with at least one risk factor for diabetes, they did not have previous diagnosis of prediabetes and diabetes mellitus and underwent oral glucose tolerance test for the diagnosis of dysglycemia. We applied the FINDRISC and the FINDRISC modified for Latin America (LA-FINDRISC) and Cuba (CUBDRISC), each with their own anthropometric parameters. The ROC curve was used to establish the cut-off point of each scale for the diagnosis of dysglycemia. Sensitivity, specificity, predictive values and likelihood ratios were calculated. The concordance between scales was calculated with Cohen's Kappa coefficient.

**Results.:**

We found that 34.5% (n=1289) of the subjects were diagnosed with dysglycemia (28.1% had prediabetes and 6.4% had type 2 diabetes without previous diagnosis). The LA-FINDRISC and CUBDRISC scales showed an almost perfect concordance with the FINDRISC scale for the different cut-off values from 11 to 16 (0.882-0.890 and 0.910-0.922, respectively). The optimal cutoff point for detecting persons with dysglycemia was ≥ 13 for the FINDRISC and CUBDRISC scales (sensitivity was 63.6% and 61.6%; specificity was 84.3% and 86.0%, respectively) and ≥11 for LA-FINDRISC (sensitivity 58.0% and specificity 88.0%).

**Conclusions.:**

We found almost perfect concordance between the diabetes risk scales. The FINDRISC score modified for Cuba proved to be a useful tool to identify persons with prediabetes and diabetes with a cut-off point of 13 in a Cuban population.

## INTRODUCTION

Diabetes mellitus (DM) is a heterogeneous syndrome characterized by chronic hyperglycemia and disorders in glucose, lipid and protein metabolism, as a consequence of an absolute or relative deficit in insulin secretion or insulin resistance ^1^^,^^2^.

The prevalence of DM has increased considerably in the world ^3^. In Central and South America, it is estimated to reach a prevalence rate of 9.5% ^3^. In the 2021 statistical yearbook of the Cuban Ministry of Public Health, a DM prevalence rate of 66.9 per 1000 inhabitants was reported, three times higher than 20 years ago ^4^.

Undiagnosed diabetes and prediabetes are a major concern because of the high risk of developing chronic complications and the resulting increased costs associated with them. The atlas of the International Diabetes Federation (IDF) in its tenth edition of 2021 indicates that in the world population aged 20 to 79 years, 537 million adults suffer from DM (10.5%), 240 million have undiagnosed DM (44.7%) out of the subjects with DM, 541 million have altered glucose tolerance (AGT) (10.6%) and 319 million show altered fasting glucose (AFG) (6.2%), with risk of developing diabetes ^5^. In the Central American and Caribbean region, 32.8% of DM cases are undiagnosed ^5^. In Cuba, according to the III National Survey of Risk Factors, 42.0% of the population had unknown diabetes ^6^.

Interest in prediabetes has increased in recent years because of its importance as a metabolic status and predisposing condition for future progression to DM and atherosclerotic cardiovascular disease, in addition to the fact that it carries a high probability of developing many of the complications normally associated with this disease, such as diabetic retinopathy, peripheral neuropathy, diabetic renal disease and macrovascular complications ^2^^,^^7^^,^^8^.

Currently, there is no worldwide consensus on the strategy for the detection of DM and prediabetes. Fasting blood glucose is the most commonly used test for this purpose; however, the oral glucose tolerance test (OGTT) is the most specific test for the diagnosis of DM and prediabetes. Glycosylated hemoglobin (HbA1c) is also recommended by some expert committees for the diagnosis of this disease ^3^^,^^7^.

For the identification of subjects at risk for type 2 diabetes, simple, quick, inexpensive, and noninvasive scoring questionnaires are available, which could reduce the number of people who have to undergo OGTT ^7^^,^^9^^-^^11^. 

Screening for type 2 diabetes mellitus (DM2) and prediabetes, with appropriate interventions, appears to be cost-effective ^9^. The Finnish Diabetes Risk Score (FINDRISC) is one of the most effective and widely used screening tools to detect new cases of DM2 and prediabetes ^10^^-^^12^. However, FINDRISC needs to be validated in populations other than the original Finnish population in order to determine performance attributes (sensitivity and specificity) ^13^.

FINDRISC is available in almost all European languages (including Spanish), has been validated in most European populations ^10^^,^^11^^,^^14^^-^^21^ and Spanish-speaking countries ^22^, which includes Colombia ^24^^-^^26^, Peru ^27^, Venezuela ^28^^,^^29^, Mexico ^30^^,^^31^, Spain ^16^^,^^20^ and recently Argentina ^12^.

The FINDRISC scale has been modified and used in different Latin American countries, being called LA-FINDRISC ^26^^,^^28^^,^^29^. The first time, it was used in a Colombian population, on the basis that waist circumference values are higher in this population and that abdominal obesity is considered a risk factor for diabetes, the score of this variable was the only one that was modified in the FINDRISC, defining ≥ 90 in women and ≥ 94 in men as the cut-off point ^13^^,^^26^. The results of the validations of the FINDRISC and LA-FINDRISC questionnaires showed that there are different cut-off values for the score with which the risk of dysglycemia can be predicted, depending on the context of the countries. The cut-off point for identifying people with dysglycemia in Europe ranged from 9 to 15 points ^11^^,^^14^^-^^18^^,^^20^^,^^32^, while in Latin America it ranged from 9 to 14 points ^12^^,^^24^^-^^26^^,^^29^^-^^31^.

During the literature review, we found that, in Cuba, the FINDRISC scale has been applied in small samples of the populations of Cienfuegos and Pinar del Río but without defining the optimal cut-off value to identify people with dysglycemia ^33^^,^^34^. In the two publications mentioned above, only the distribution of the different diabetes risk scores in each population was analyzed.

In this research, we used a modified version of the FINDRISC scale for Cuba (CUBDRISC: CUBan Diabetes RIsk SCore) in which the abdominal obesity waist circumference values for Europe were replaced by the cut-off point for Cubans ^35^. As there are no previous studies in Cuba that have evaluated the usefulness of the LA-FINDRISC and CUBDRISC scales as screening tools for glucose regulation disorders, we decided to conduct this research, that aimed to evaluate the modified FINDRISC for Cuba (CUBDRISC) as a tool for the detection of persons with dysglycemia (undiagnosed diabetes and prediabetes) in a Cuban population and to compare the performance of this score with the FINDRISC and LA-FINDRISC scales.

**KEY MESSAGES**

Motivation for the study. In Ecuador, foodborne disease (FBD) incidence rates adjusted for population size have not been estimated, which will serve to identify priority geographic areas.

Main findings. Between 2015-2020, 113,695 cases of FBD were identified, with “other food poisoning” and hepatitis A being most common. The highest incidence rates were found in the Amazon region. There is marked variability by geographic region in the incidence rates during the study period.

Public health implications. It is necessary to optimize the registry system, establish detection and treatment protocols, analyze the causes related to the higher incidence of FBD in the Amazon region, and design a health promotion program focused on preventing contamination and establishing diagnostic and treatment protocols.

## MATERIAL AND METHODS

### Population and study design

This was an epidemiological, cross-sectional, analytical and secondary source (database) study in persons with at least one risk factor for diabetes ^2^^,^^7^, based on the database of a dysglycemia screening study in the general population conducted in the municipality of Jaruco in 2008-2012 ^36^. Jaruco is a municipality in the province of Mayabeque in Cuba, located about 30 km east of Havana. It has a territorial extension of 275.7 km². In addition to the town of Jaruco (approximately 9,000 inhabitants), the municipality includes the urban towns of San Antonio de Río Blanco, Caraballo and Bainoa.

We interviewed 9056 adult persons aged ≥ 20 years (mean: 53.0 and SD: 16.5) from 23 clinics in the town of Jaruco, 905 individuals were discarded due to the absence of diabetes risk factors, 8151 adults were identified with at least one diabetes risk factor ^2^^,^^7^, of these 505 had known diabetes, who were excluded. We did not examine 3909 of the individuals for unforeseen logistical reasons, and finally we analyzed 3737 individuals with no previous diagnosis of prediabetes or type 2 diabetes.

Individuals with at least one of the following risk factors for diabetes ^2^ were included: Age ≥ 45 years, overweight or obese adults (BMI ≥ 25 kg/m2), family history of diabetes in the first and second degree, waist circumference ≥ 80 cm in women and ≥ 90 cm in men, obstetric history of gestational diabetes, or children weighing > 4 kg at birth, coronary ischemic or vascular disease of atherosclerotic origin, arterial hypertension, triglycerides ≥ 150 mg/dL (1.7 mmol/L), HDL cholesterol < 40 mg/dL (1.03 mmol/L), low birth weight or macrosomia, sedentary lifestyle (< 150 minutes of physical activity/week), polycystic ovary syndrome, and Acanthosis *nigricans*.

People suffering from an acute illness, evident mental incapacity to give reliable information, pregnant, with diabetes or in treatment with drugs that modify glycemia were excluded. In addition to any of the endocrine diseases associated with diabetes (Cushing’s syndrome, hyperthyroidism, pheochromocytoma, glucagonoma, acromegaly) or suspected insulinoma.

### Definitions

All participants underwent OGTT, defined as the gold standard method for the diagnosis of prediabetes and DM2 ^2^^,^^7^^,^^8^.

The diagnostic criteria for DM2 and prediabetes, altered fasting glucose (AFG) and altered glucose tolerance (AGT) from the 2012 Cuban guidelines were used for the classification, of the 2019 ALAD and the 2024 ADA ^2^^,^^7^^,^^8^, defining AFG with fasting blood glucose values between ≥ 5.6 mmol/L (100 mg/dL) and < 7 mmol/L (126 mg/dL) and at 2 hours < 7.8 mmol/L (140 mg/dL). AGT was defined with fasting blood glucose values < 5.6 mmol/L (100 mg/dL) and at 2 hours ≥ 7.8 (140 mg/dL) and < 11.1 mmol/L (200 mg/dL). Mixed or double prediabetes was defined with fasting blood glucose values ≥ 5.6 mmol/L (100 mg/dL) and < 7 mmol/L (126 mg/dL) and at 2 hours ≥ 7.8 (140 mg/dL) and < 11.1 mmol/L (200 mg/dL) and DM2 fasting blood glucose values ≥ 7.0 mmol/L (126 mg/dL) and/or at 2 hours ≥ 11.1 mmol/L (200 mg/dL) ^2^^,^^7^^,^^8^.

The term “prediabetes” includes the presence of altered fasting blood glucose (AFG), altered glucose tolerance (AGT) or both conditions at the same time (AFG + AGT) ^7^^,^^8^, all of which imply a high risk of developing DM2 and cardiovascular complications.

For this study, we used the term “dysglycemia” to refer to metabolic states that meet the criteria for diabetes, some type of prediabetes or previously undiagnosed diabetes ^11^.

### Study variables

The subjects’ weight, height, waist circumference (WC) and body mass index (BMI) were measured. WC was measured with a measuring tape with the subject standing upright, in expiration, with the abdomen relaxed, taking as reference the midpoint between the lower edge of the last rib and the anterosuperior iliac spine on each side. In cases of pendulous abdomens, WC was measured at the most prominent point of the abdomen.

Blood pressure (BP) was measured in each subject with a sphygmomanometer with a cuff according to the size of the arm. Previously, the subject was seated at rest for ten minutes. The procedure was performed three times on the subject’s right arm, with a five-minute interval. The final BP value corresponded to the average of the three measurements obtained.

Each individual underwent laboratory testing at the time of the first (baseline) blood draw, after approximately 8-12 hours of fasting. Each individual underwent the oral glucose tolerance test (OGTT), with fasting blood glucose measurements (venous plasma) and at 2 hours after an oral overload with 75.0 g of anhydrous glucose. The following laboratory data were used: fasting and 2-hour glucose values after OGTT, as well as cholesterol and triglycerides.

The information was extracted from a database from the Jaruco epidemiological study, which was created for the active screening of DM2 in the population of that locality and prepared by one of the co-authors ^36^. In addition, the variables of age, sex, anthropometric measurements (weight, height, BMI and WC) and BP or medication for the treatment of hypertension were obtained, as well as family history of DM (first and second degree), known high blood glucose previously reported at the time the questionnaire was applied and daily consumption of vegetables and fruits. Likewise, information regarding physical activity (150 minutes per week) was collected.

### Original FINDRISC questionnaire

The FINDRISC is a simple diabetes risk scoring tool originally developed in Finland to predict the incidence of diabetes among the Finnish population aged 35-64 years ^10^. It is based on eight simple diabetes risk factors, such as age (years), BMI (kg/m^2^), waist circumference (cm), history of high blood pressure, history of raised blood glucose, family history of diabetes, daily fruit or vegetable consumption, and daily physical activity ^10^. The tool does not require laboratory testing and has different scores weighted according to the associated risk, with a final score ranging from 0 to 26 ^10^.

The level of risk for diabetes is evaluated in five categories ^10^: less than 7 points (low risk, 1% will develop DM2); between 7-11 points (slightly elevated, 4% will develop DM2); between 12-14 points (moderate risk, 17% will develop DM2); between 15-19 points (high risk, 33% will develop DM2); greater than 20 points (very high risk, 50% will develop DM2).

The FINDRISC questionnaire was used with the data on the variables mentioned above for each individual. The variables and their scores are described in Table 1.

**Table 1:** Variables of the FINDRISC questionnaires ^10^ and those modified for Latin America (LA-FINDRISC) ^30^ and for Cuba (CUBDRISC) ^36^ with their respective scores.

				Score
1) Age				
	Less than 45 years old			0 points
	45 to 54 years old			2 points
	55 to 64 years old			3 points
	Over 64 years old			4 points
2) Body mass index				
	Lower than 25 Kg\m^2^			0 points
	25 - 30 Kg\m^2^			1 point
	Higher than 30 Kg\m^2^			3 points
3) Waist circumference				
	FINDRISC ^10^	LA-FINDRISC ^30^	CUBDRISC ^36^	
	M: lower 94 cm	M: lower 94 cm	M: lower 90 cm	0 points
	W: lower 80 cm	W: lower 90 cm	W: lower 80 cm	
	M: 94-102 cm	-	-	3 points
	W: 80-88 cm	-	-	
	M: higher than 102 cm	M: ≥94 cm	M: ≥90 cm	4 points
	W: higher than 88 cm	W: ≥90 cm	W: ≥80 cm	
4) Do you usually engage in physical activity for 30 minutes per day?				
	Yes			0 points
	No			2 points
5) How often do you eat vegetables or fruits?				
	Daily			0 points
	Not daily			1 point
6) Have you ever taken antihypertensive medications on a regular basis?				
	No			0 points
	Yes			2 points
7) Have you ever had high blood glucose levels (during a check-up, pregnancy, or a day when you were sick)?				
	No			0 points
	Yes			5 points
8) Has any member of your family been diagnosed with type 1 or type 2 diabetes?				
	No			0 points
	Yes (grandparents, uncles, aunts, cousins, nieces, nephews, wife with GD)			3 points
	Yes (parents, siblings, children)			5 points
The risk of developing diabetes mellitus in 10 years is:				
	Total score:	Risk of developing diabetes:	Risk level interpretation:	
	Less than 7 points	1%	Low	
	7-11 points	4%	Slightly elevated	
	12-14 points	17%	Moderate	
	15-20 points	33%	High	
	Over 20 points	50%	Very high	

M: men, W: women, GD: gestational diabetes.

### Modified FINDRISC for Latin America and Cuba

The modified LA-FINDRISC ^28^^,^^29^^)^ and CUBDRISC questionnaires were also used for each individual and similarly consist of eight variables: age, BMI, WC, physical activity, daily vegetable and fruit consumption, use of antihypertensive drugs, personal history of hyperglycemia, and family history of diabetes. However, the WC cut points were adjusted for Latin America (WC ≥ 94 cm for men and ≥ 90 cm for women) ^13^^,^^29^^)^ and for Cuba (WC ≥ 90 cm for men and ≥ 80 cm for women) ^35^, adding 4 points for subjects with abdominal obesity and no points for those with lower values of the WC cut points ^28^^,^^29^, the total score ranging from 0 to 26 points. See modified scales of risk of developing DM2 in 10 years (Table 1).

### Statistical analysis

For the descriptive analysis, the qualitative variables included in the questionnaires [age, BMI and waist circumference categorized, practice or not of physical activity, daily consumption or not of vegetables and fruits, treatment or not for AHT, history or not of hyperglycemia and history or not of family members with diabetes or gestational diabetes, with the presence (or not) of dysglycemia in general, prediabetes and diabetes in particular] were expressed in absolute frequency and their respective percentages (%). The Chi-square test was used to compare proportions.

The predictive power and performance of the FINDRISC, LA-FINDRISC and CUBDRISC scales in the detection of dysglycemia (unknown DM2 and prediabetes) was determined by calculating sensitivity, specificity, positive predictive value (PPV), negative predictive value (NPV) and the area under the receiver operating characteristic curve (AUC-ROC). The variable was considered to have good discriminatory power when the area under the ROC curve was different from 0.5 (p < 0.05 and confidence interval not containing 0.5). A perfect diagnostic test has an AUC of 1.0. The accuracy of a test depends on the AUC-ROC (AUC 0.9-1.0: excellent; 0.8-0.9: very good; 0.7-0.8: good; 0.6-0.7: sufficient; 0.5-0.6: poor; <0.5 test not useful) ^37^.

Cut-off points were calculated for each questionnaire from score 11 to 16. Likelihood ratios were also calculated to determine the clinical utility of the diagnostic test for the different cut-off points of the used scales (FINDRISC, LA-FINDRISC and CUBDRISC). The respective 95% confidence intervals (95%CI) were calculated. The predictive capacity of each questionnaire was analyzed when comparing non-dysglycemic versus dysglycemic individuals (prediabetes and detected diabetes) and by sex, as well as between prediabetes versus individuals with normal glucose tolerance and diabetes versus individuals with normal glucose tolerance.

The likelihood ratio (LR) was used to identify the score with the best discriminatory ability based on estimates of AUC-ROC, specificity, and sensitivity ^38^. A positive likelihood ratio (LR+) > 2 and a negative likelihood ratio < 0.5 were considered useful.

The degree of diagnostic agreement of the cut-off values obtained between the modified scales (LA-FINDRISC and CUBDRISC) with the original FINDRISC scale (gold standard) was calculated by Cohen’s kappa coefficient. The kappa coefficient was interpreted on the basis of the six levels of concordance strength proposed by Landis and Koch ^39^: ≤ 0.00 (poor); 0.01-0.20 (slight); 0.21-0.40 (acceptable), 0.41-0.60 (moderate); 0.61-0.80 (considerable) and 0.81-1.00 (almost perfect). P values less than 0.05 were accepted as statistical significance values.

The SPSS for Windows® version 19.0 and Epidat version 3.1 statistical software were used for the data analysis.

### Ethical aspects

The Jaruco study database was used for this research, and the confidentiality of the participants was preserved. The data analysis was approved by the Research Ethics Committee (CEI) of the Institute of Endocrinology (INEN) (Code: I070LH2304, October 7, 2021). The study has the authorization of the principal researcher of the original study. The participating researchers assume the commitment of honesty in the analysis and reporting of the results.

## RESULTS

### Demographic, anthropometric, clinical and biochemical characteristics of the participants

We studied 3737 people (supplementary material) with some risk factor for diabetes mellitus of whom those older than 45 years (65.3%) predominated over those younger than 45 years (34.7%). However, when distributed by different age groups, we found differences between men and women (p=0.003; Table 2). Females predominated over males (Table 3). There was a predominance of people with white skin followed by those with mestizo and black skin, respectively. According to BMI, 35.9% were overweight and 17.6% had obesity; respectively, in addition, 67.6% of the individuals presented altered abdominal circumference and 47.5% of the subjects had AHT or treatment. Women had a higher proportion of obesity, abdominal obesity and AHT than men (Table 2).

**Table 2:** Demographic, anthropometric, clinical and biochemical characteristics in persons with at least one risk factor for type 2 diabetes mellitus.

Clinical and biochemical anthropometric characteristics		Total n (%) n=3737	Women n (%) n=2194	Men n (%) n=1543
Age				
	20 - 39 years	866 (23.2)	542 (24.7)	324 (21.0)
	40 - 59 years	1545 (41.3)	923 (42.1)	622 (40.3)
	60 - 79 years	1086 (29.1)	595 (27.1)	491 (31.8) ^a^
	≥ 80 years	240 (6.4)	134 (6.1)	106 (6.9)
Skin color				
	White	2970 (79.5)	1375 (79.1)	1235 (80.0)
	Black	363 (9.7)	219 (10.0)	144 (9.3)
	Mestizo	404 (10.8)	240 (10.9)	164 (10.6)
Body mass index				
	Normal weight or underweight	1737 (46.5)	948 (43.2)	789 (51.1)
	Overweight	1341 (35.9)	792 (36.1)	549 (35.6)
	Obesity	659 (17.6)	454 (20.7) ^b^	205 (13.3)
	Waist circumference (M ≥ 90 cm and W ≥ 80 cm)	2528 (67.6)	1660 (75.7) ^b^	868 (56.3)
	Arterial hypertension (≥130/85 mmHg or treatment)	1774 (47.5)	1037 (52.7) ^b^	737 (47.8)
Dysglycemia				
	Diabetes detected	238 (6.4)	135 (6.2)	103 (6.7)
	AFG	798 (21.4)	425 (19.4)	373 (24.2) ^c^
	AGT	132 (3.5)	81 (3.7)	51 (3.3)
	AFG + AGT	121 (3.2)	65 (3.0)	56 (3.6)
	No Dysglycemia	2448 (65.5)	1488 (67.8)	960 (62.2)
	Cholesterol ≥ 5.2 mmol/L (200 mg/dL)	978^e^ (26.2)	635 ^g^ (28.9) ^b^	343 ^h^ (22.2)
	Triglycerides ≥ 1.7 mmol/L (150 mg/dL)	1050 ^f^ (28.1)	565 ^g^ (25.8)	485 ^i^ (31.4) ^d^

a p= 0.003 between age groups by sex, ^b^ p<0.0001 vs male ^c^ p=0.0005 vs female ^d^ p=0.0002 vs female ^e^ n=3732, ^f^ n=3734, ^g^ n=2193, ^h^ n=1539, ^i^ n=1541. Chi-square test was used for all variables.

AFG: altered fasting glucose, AGT: altered glucose tolerance, AFG + AGT: both conditions at the same time, M: men, W: women.

**Table 3:** Frequencies of the variables of the original questionnaire (FINDRISC) in persons with at least one risk factor for type 2 diabetes mellitus.

Variable		n (%)
Age (years)		
	20-44	1295 (34.7)
	45-54	744 (19.9)
	55-64	722 (19.3)
	≥65	976 (26.1)
Sex		
	Men	2194 (58.7)
	Women	1543 (41.3)
Body mass index		
	Underweight or normal weight	1737 (46.5)
	Overweight	1341 (35.9)
	Obesity	659 (17.6)
Waist circumference (cm)		
	No abdominal obesity (M <94 and W <80)	1421 (38.0)
	Mild abdominal obesity (M 94-102 and W 80-88)	1116 (29.9)
	Severe abdominal obesity (M >102 and W >88)	1200 (32.1)
Physical activity ^a^		
	Yes	1990 (53.3)
	No	1747 (46.7)
Consumes vegetables and fruits ^b^		
	Yes	649 (17.4)
	No	3088 (82.6)
Has had HTA or treatment		
	Yes	1774 (47.5)
	No	1963 (52.5)
History of high blood glucose		
	Yes	1326 (35.5)
	No	2411 (64.5)
History of first-degree relatives with diabetes or gestational diabetes		
	Yes	1116 (29.9)
	No	2621 (70.1)

M: men, W: women

a Performs physical activity at least 30 minutes a day, ^b^ Consumes vegetables and/or fruits daily.

Regarding the level of education, 8.8% of the adults had university level education (329/3737), 17.8% (667/373737) pre-university, 12.9% (483/373737) technical and 38.5% high school education (1438/3737).

Prediabetes (AFG, AGT or AFG/AGT) was identified in 28.1% and 6.4% of those with detected DM2. We found that men had a higher frequency of AFG than women (p=0.0005) (Table 2). We also found that 28.1% of the individuals had hypertriglyceridemia and 26.2% had hypercholesterolemia. The highest percentages of hypercholesterolemia and hypertriglyceridemia were found in women and men, respectively (Table 2).

### Frequencies of the variables in the original questionnaire (FINDRISC)

In the study sample, 34.7% of the subjects were under 45 years of age and in the rest of the age groups the frequency decreased discretely (Table 3). According to waist circumference, 38.0% of the subjects were found to have no abdominal obesity; 29.9% had mild abdominal obesity and 32.1% had severe abdominal obesity (Table 3).

We found that 53.3% of the participants were physically active and 17.4% consumed vegetables and fruits daily. Of those evaluated, 47.5% were hypertensive or took antihypertensive drugs. In addition, 29.9% had a first-degree family history of diabetes or gestational diabetes. Finally, 35.5% had ever presented high blood glucose levels (Table 3).

### Diagnostic concordance of the various cut-off points according to the studied questionnaires

The modified LA-FINDRISC and CUBDRISC scales showed an almost perfect degree of agreement with the original FINDRISC scale for the various cut-off values from 11 to 16 (0.882-0.890 and 0.910-0.922, respectively) (Table 4).

**Table 4:** Diagnostic concordance of the different cut-off points of the original FINDRISC scale with the modified scales (LA-FINDRISC and CUBDRISC).

	Modified scales	Kappa	Standard error	Significance	Degree of concordance
FINDRISC ≥ 11 p	LA-FINDRISC	0.882	0.008	<0.0001	Almost perfect
	CUBDRISC	0.910	0.007	<0.0001	Almost perfect
FINDRISC ≥ 12 p	LA-FINDRISC	0.893	0.007	<0.0001	Almost perfect
	CUBDRISC	0.915	0.007	<0.0001	Almost perfect
FINDRISC ≥ 13 p	LA-FINDRISC	0.904	0.007	<0.0001	Almost perfect
	CUBDRISC	0.921	0.006	<0.0001	Almost perfect
FINDRISC ≥ 14 p	LA-FINDRISC	0.907	0.007	<0.0001	Almost perfect
	CUBDRISC	0.933	0.006	<0.0001	Almost perfect
FINDRISC ≥ 15 p	LA-FINDRISC	0.888	0.009	<0.0001	Almost perfect
	CUBDRISC	0.924	0.007	<0.0001	Almost perfect
FINDRISC ≥ 16 p	LA-FINDRISC	0.890	0.009	<0.0001	Almost perfect
	CUBDRISC	0.922	0.007	<0.0001	Almost perfect

Diagnostic concordance: kappa coefficient ≤ 0.00 (poor); 0.01-0.20 (slight); 0.21-0.40 (acceptable), 0.41-0.60 (moderate); 0.61-0.80 (considerable) and 0.81-1.00 (almost perfect).

### Cut-off points for identifying persons with dysglycemia according to the questionnaires

The optimal cutoff point was ≥ 13 regarding the ability of the FINDRISC and CUBDRISC questionnaires to identify persons with dysglycemias. In contrast, the optimal cutoff point was ≥ 11 to detect persons with dysglycemias for the LA-FINDRISC scale (Table 5).

**Table 5:** Comparison of non-diglycemic vs. dysglycemic individuals (prediabetes and detected diabetes) according to various cut-off points of the questionnaires analyzed.

Scale	Cut-off points	S 95%IC	E 95%IC	PPV 95%IC	NPV 95%IC	LR+ 95%IC	LR- IC95%	AUC 95%IC	p-value
FINDRISC	≥ 11	55.7 53.5-57.9	89.0 87.6-90.5	85.0 83.0-87.0	64.4 62.5-66.3	5.1 4.4-5.8	0.50 0.47-0.52	0.745 0.733-0.760	
LA-FINDRISC		58.0 55.7-60.3	88.0 86.5-89.5	82.2 80.1-84.4	68.7 66.8-70.5	4.8 4.3-5.5	0.48 0.45-0.50	0.755 0.741-0.768	<0.0001
CUBDRISC		53.4 51.2-55.5	90.6 89.2-92.1	88.4 86.6-90.2	59.4 57.4-61.3	5.7 4.9-6.7	0.41 0.49-0.54	0.739 0.726-0.752	
FINDRISC	≥ 12	59.1 56.7-61.4	86.4 84.9-87.9	78.7 76.5-81.0	71.2 69.4-73.0	4.4 3.9-4.9	0.47 0.45-0.50	0.750 0.736-0.764	
LA-FINDRISC		60.7 58.3-63.2	84.9 83.4-86.4	74.8 72.4-77.2	74.6 72.8-76.3	4.0 3.6-4.5	0.46 0.43-0.49	0.747 0.732-0.761	<0.0001
CUBDRISC		56.3 54.1-58.6	87.6 86.1-89.1	82.1 80.0-84.2	66.5 64.6-68.4	4.5 4.0-5.2	0.50 0.47-0.53	0.743 0.729-0.757	
FINDRISC	≥ 13	63.6 61.1-66.1	84.3 82.8-85.8	72.3 69.8-74.8	78.2 76.5-79.8	4.0 3.7-4.5	0.43 0.40-0.46	0.753 0.738-0.767	
LA-FINDRISC		65.3 62.7-67.9	83.0 81.5-84.5	68.5 66.0-71.1	80.8 79.3-82.4	3.8 3.5-4.2	0.42 0.39-0.45	0.747 0.732-0.762	<0.0001
CUBDRISC		61.6 59.2-64.0	86.0 84.5-87.5	77.0 74.6-79.3	74.8 73.0-76.5	4.4 3.9-4.9	0.45 0.42-0,.48	0.759 0.744-0.773	
FINDRISC	≥ 14	68.1 65.4-70.7	82.1 80.6-83.6	65.3 62.7-68.0	83.9 82.4-85.3	3.8 3.5-4.2	0.39 0.36-0.42	0.745 0.731-0.761	
LA-FINDRISC		69.2 66.5-72.0	80.6 79.1-82.2	60.9 58.2-63.6	85.7 84.3-87.2	3.6 3.3-3.9	038 0.35-0.42	0.733 0.718-0.748	<0.0001
CUBDRISC		66.4 63.8-68.9	83.5 82.0-85.0	69.5 67.0-72.1	81.5 80.0-83.0	4.0 3.7-4.5	0.40 0.37-0.43	0.755 0.740-0.770	
FINDRISC	≥ 15	72.2 69.5-75.0	80.0 78.5-81.5	58.1 55.4-60.8	88.2 86.9-89.5	3.6 3.3-3.9	0.35 0.31-0.38	0.732 0.717-0.747	
LA-FINDRISC		74.4 71.6-77.3	79.0 77.4-80.5	54.4 51.6-57.1	90.2 89.0-91.4	3.5 3.3-3.8	0.32 0.29-0.36	0.723 0.708-0.738	<0.0001
CUBDRISC		70.5 67.8-73.2	81.6 80.1-83.1	63.2 60.5-65.8	86.1 84.7-87.5	3.8 3.5-4.2	0.36 0.33-0.40	0.746 0.731-0.761	
FINDRISC	≥ 16	76.7 73.8-79.7	77.6 76.1-79.2	49.7 46.9-52.4	92.1 91.0-93.2	3.4 3.2-3.7	0.30 0.26-0.34	0.829 0.812-0.845	
LA-FINDRISC		79.2 76.3-82.2	76.9 75.3-78.4	46.5 43.7-49.2	93.4 92.6-94.6	3.4 3.2-3.7	0.27 0.23-0.31	0.700 0.686-0.715	<0.0001
CUBDRISC		75.2 72.4-78.0	79.2 77.7-80.7	54.9 52.1-57.6	90.5 89.3-91.7	3.6 3.4-3.9	0.31 0.28-0.35	0.727 0.712-0.741	

S: sensitivity, E: specificity, PPV: positive predictive value, NPV: negative predictive value. 95%CI: 95% confidence interval, LR+: positive likelihood ratio, LR-: negative likelihood ratio, AUC: area under the curve.

When analyzing the frequency of dysglycemia in the subjects according to the presence or not of the optimal cutoff point ≥ 13 for the FINDRISC and CUBDRISC scales, we noticed that persons with this score had a higher proportion of dysglycemia (prediabetes or DM2) (63.6% and 61.6%, respectively) than those with the score lower than 13 (15.7% and 13.9%, respectively) (p<0.0001). However, according to the presence or not of the cutoff point ≥ 11 for the LA-FINDRISC scale, we found participants that with that score had a higher proportion of dysglycemia (58.0%) than those with the score less than 11 (12.0%) (p<0.0001) (data not shown).

### Area under the curve and optimal cut-off points for the detection of prediabetes or unknown diabetes

**Figure 1:**
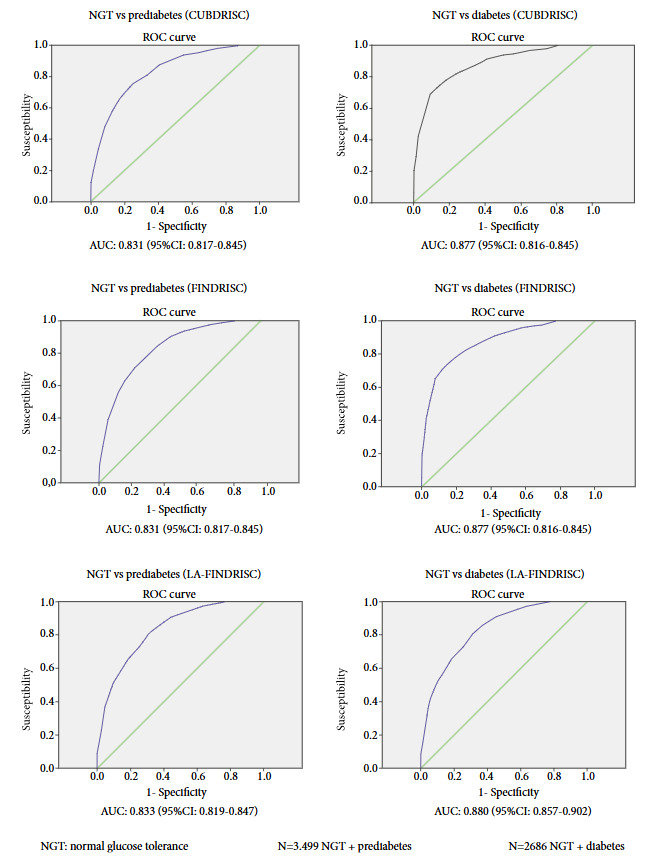
Area under the curve (AUC) for the detection of prediabetes or unknown diabetes compared to persons without dysglycemia when applying the different scales.

Figure 1 shows the separate AUC-ROC curves for detection of previously undiagnosed diabetes and prediabetes in the analyzed sample. The AUC for prediabetes was 0.831 (95% CI: 0.817-0.845) for the FINDRISC and CUBDRISC questionnaires, whereas it was 0.877 for diabetes screening (95% CI: 0.817-0.845) for FINDRISC and 0.877 (95% CI: 0.816-0.845) for CUBDRISC. In contrast, the AUC was 0.833 (95% CI: 0.819-0.847) for the LA-FINDRISC scale identification of prediabetes and it was 0.880 (95% CI: 0.857-0.902) for diabetes.

The optimal cutoff point for the FINDRISC and CUBDRISC scales to identify prediabetes was ≥ 12 points and for diabetes it was ≥ 13 and ≥ 14 points, respectively. In contrast for the LA-FINDRISC scale for prediabetes the best cutoff point was ≥ 11 points and for diabetes it was ≥ 12 points (data not shown).

### Optimal cutoff points for identifying persons with dysglycemia according to sex

The optimal cutoff point for identifying persons with dysglycemias on the FINDRISC and CUBDRISC scales for men was ≥11 points (sensitivity= 67.5% and 62.3%, specificity= 86.3% and 89.0%, positive likelihood ratio of 4.9 and 5.7, AUC of 0.783 and 0.772, respectively) and ≥13 points for women (sensitivity=57.9%, specificity=88.4%, positive likelihood ratio of 5.0 and AUC of 0.761) predicted dysglycemia in the subsamples studied using the FINDRISC scale. In contrast, the CUBDRISC scale with a cutoff point of ≥14 points identified women with dysglycemia (sensitivity= 61.6%, specificity= 86.7%, positive likelihood ratio of 4.6 and AUC 0.763). The optimal cutoff points for men and women for LA-FINDRISC was 11 for both sexes (sensitivity= 65.2% and 53.3%, specificity= 86.7% and 89.0, positive likelihood index of 4.9 and 4.8, AUC 0.775 and 0.742, respectively) (data not shown).

## DISCUSSION

The high prevalence and incidence of DM2 created the need to develop screening tools to diagnose and predict the risk of the disease worldwide ^3^^,^^5^. Of all the studied questionnaires, the FINDRISC is possibly the most widely accepted, and has been used in population-based intervention studies mainly in Europe ^10^^-^^12^^,^^15^^-^^23^^,^^33^, Latin America ^24^^,^^25^^,^^27^^-^^30^^,^^32^, and the modified one for Latin America (LA-FINDRISC) ^26^^-^^30^.

Intervention toward DM2, because of its high prevalence and its short- and long-term complications, can be achieved by prevention and early prediction of dysglycemia ^11^^,^^13^^,^^15^^,^^16^^,^^33^^,^^40^^-^^43^. Like other population screening studies, we have chosen the FINDRISC ^10^^-^^12^^,^^20^^-^^23^^,^^25^^,^^28^^-^^30^ and LA-FINDRISC ^26^^-^^30^ questionnaires because of the ease of obtaining demographic, anthropometric, clinical, and family history parameters to identify individuals with dysglycemia ^11^^,^^12^^,^^14^^-^^16^^,^^19^^,^^21^^,^^26^^,^^29^^-^^31^^,^^33^.

This is the first study adapted to the Cuban population in which the risk score for diabetes and prediabetes is calculated for the FINDRISC, LA-FINDRISC and the modified scale for Cuba (CUBDRISC). Validating the CUBDRISC scale for the Cuban population is an important task because the optimal scores for each of the evaluated items are variable for each population ^13^.

In a multivariate analysis including all parameters of the original FINDRISC cohort, an age > 45 years increased the risk of DM2 ^10^. In our study, most subjects were aged ≥ 45 years (n=2442), so the age-associated risk was higher.

In their study, Naranjo *et al*. ^35^ included a high proportion of overweight and obese individuals (80.6%). The proportion of overweight individuals in the aforementioned study was higher than in our study (54.5%). In this study, women showed a higher frequency of obesity compared to men (20.7% vs. 13.3%, respectively). Nevertheless, the frequency of abdominal obesity according to FINDRISC categories between our investigation and that of Naranjo *et al*. were similar when analyzing both sexes ^35^. However, women had a higher proportion of abdominal obesity (≥ 80 cm) ^36^ relative to men (≥ 90 cm) ^36^ (75.7% and 56.3%, respectively).

The high proportion of women with hypertension in this study must be the result of the increased frequency of obesity in women compared to men. These data are different from those found in the National Survey of Risk Factors, in which there were no differences in the prevalence of hypertension by sex ^6^.

The FINDRISC questionnaire includes daily consumption of fruits and vegetables, although this variable contributed very little to the predictive power of diabetes in the FINDRISC study ^10^. The fact that most individuals did not consume fruits and vegetables on a daily basis underscores the importance of addressing this factor in DM2 prevention programs. A previous study conducted in primary care in Pinar del Rio, also reported a low daily consumption of fruits and vegetables ^35^. In contrast, several studies ^10^^,^^11^^,^^25^ that included this variable, reported that the daily consumption of fruits and vegetables was higher than in our study and that of Naranjo *et al*. ^35^ However, it was similar to a Venezuelan study ^30^.

We found 6.4% of individuals with detected DM2, as well as 28.1% of individuals with prediabetes, using the OGTT as a diagnostic test. In total, 1289 (34.5%) patients presented either of the two aforementioned conditions. The frequency of altered glucose metabolism in this research was slightly higher than that found in primary care in a multicenter study in Europe (30.7%; 3512/11444) ^11^. The aforementioned is reasonable since this research included people with at least one risk factor for diabetes ^2^^,^^7^^,^^8^.

Regarding the frequency of prediabetes (AGT or AFG/AGT) and DM2 detected, we did not obtain differences in relation to sex, except in AFG, where the male sex prevailed with respect to the female sex (24.2% vs 19.4%; respectively), this is incongruent with most studies carried out in Europe with a higher prevalence of DM2, in which the male sex predominated ^10^^,^^11^. On the other hand, the similar frequency of DM2 detected in both sexes does not coincide with that reported in Cuba, where the highest prevalence of DM2 was found in the female sex ^4^.

The proportion of detected DM2 (n=238) and prediabetes (1051) in this study suggests a substantial increase in the prevalence of DM2 in the next 10 years, if effective preventive measures are not taken. We found that having an individual risk of moderate (12-14 points), high (15-20 points) to very high (more than 20 points), increases the frequency of people with dysglycemia. Several studies have found similar results ^12^^,^^17^^,^^44^.

Taking into account the different types of prediabetes and DM2, we found that participants with prediabetes and DM2 had a score ≥ 12 with the following proportions for the different diabetes risk scales (FINDRISC, LA-FINDRISC and CUBDRISC): FINDRISC [AFG 74.9%; AGT 78.0%, double prediabetes (AFG/AGT) 94.2% and DM2 of 84.0%]. LA-FINDRISC [AFG 70.6%; TGA 68.9%, double prediabetes (AFG / AGT) 93.4% and DM2 of 82.8%] and in the CUBDRISC [AFG 78.6%; AGT, 82.6%, double prediabetes (AFG / AGT) 95.9% and DM2 of 86.6%]. The aforementioned data from the different scales indicate that these questionnaires detect a large number of persons with dysglycemia. Several studies using the FINDRISC and LA-FINDRISC scales have agreed with our results ^12^^,^^13^^,^^25^^,^^29^^,^^31^^,^^33^. Consequently, the CUBDRISC scale detected a higher frequency of persons with AFG and AGT than the LA-FINDRISC scale (p=0.0003 and 0.0146, respectively), which is reasonable, as the waist circumference cutoff values used for the CUBDRISC scale ^36^^)^ are lower than those used in the FINDRISC ^10^^)^ and LA-FINDRISC scales ^29^^,^^30^^,^^45^.

Few studies simultaneously compare more than two diabetes risk scales (FINDRISC, LA-FINDRISC and CUBDRISC). In other populations in Latin America and Europe, similar results have been found between the different risk scales, and concordance has been found between them ^28^^,^^29^^,^^46^. Such studies have considered the LA-FINDRISC scale to be valid ^28^^,^^29^. This research revealed an almost perfect degree of agreement according to the kappa coefficient between the LA-FINDRISC and CUBDRISC scales with the original FINDRISC scale for all the cut-off points studied.

The authors suggest that the implementation of the CUBDRISC and FINDRISC in the Cuban population would be a useful alternative to detect people with dysglycemia (prediabetes and diabetes), especially in settings with limited resources, such as those where fasting blood glucose or other markers are not available ^28^. Similar results were obtained with the FINDRISC and LA-FINDRISC test, which performed adequately as a tool for the detection of dysglycemia in cross-sectional studies ^11^^,^^21^.

The FINDRISC was originally developed in a prospective cohort to identify individuals at high risk of developing DM2 ^10^, and cross-sectional studies that have analyzed the performance of this score as a screening tool for the detection of undiagnosed DM2 and prediabetes show that optimal cut points vary widely, from 9 to 15 ^11^^,^^16^^,^^18^^,^^21^^,^^30^^,^^45^^,^^47^^,^^48^.

The main difference between the analyzed scales was the different cut-off points for WC ^10^^,^^27^^,^^30^^,^^36^. The used the WC cut-off points in the CUBDRISC scale are lower ^36^^)^ providing 4 points and the FINDRISC results in a value of zero to men when WC was < 94 cm and a score of 3 to women with measurements from 80 to 88 cm ^10^. In contrast, the WC cut-off point in the LA-FINDRISC scale is much higher for both sexes, therefore, all values below 94 cm for men and 90 cm for women do not contribute points (zero) ^27^^,^^29^.

When analyzing all cases (both sexes), the optimal cutoff point was ≥ 13 to identify participants with dysglycemia in the FINDRISC and CUBDRISC questionnaires which was similar to that found by other authors ^12^^,^^21^^,^^28^^,^^45^. In contrast, the optimal cutoff point for the LA-FINDRISC questionnaire was ≥ 11, which is lower than that reported by other researchers (≥ 14) ^30^^)^ and close to that obtained by Bernabe-Ortiz *et al*. (≥ 10) ^28^^)^ and Nieto-Martínez *et al*. (≥ 9) ^29^. The use of the CUBDRISC scale could be recommended to assess the risk of dysglycemia in the Cuban population, taking into account that it uses the cut-off points of waist circumference for the Cuban population ^36^.

We consider that the optimal scores obtained in this research for the FINDRISC, LA-FINDRISC and CUBDRISC scales to detect persons with dysglycemia and diabetes increase the specificity of the survey, thus decreasing the number of false positive cases, increasing the positive likelihood ratio and also showing a better area under the curve ^16^^,^^31^. Nevertheless, we obtained an optimal cutoff point ≥ 12 points for the FINDRISC and CUBDRISC scales for the identification of subjects with prediabetes, whereas it was ≥ 11 points for the LA-FINDRISC. Regarding the optimal cut-off points for the identification of DM2 in the FINDRISC, LA-FINDRISC and CUBDRISC scales (13, 12 and 14, respectively) were slightly higher than for prediabetes. The cutoff points when employing the FINDRISC questionnaire in several studies showed close results (scores of 11, 13, 14, and 15) for the detection of persons with DM2 ^16^^,^^21^^,^^25^^,^^28^^,^^45^.

Literature presents several differences for the optimal cut-off points when using the OGTT for the diagnosis of dysglycemia, for example; Villena *et al*. ^45^^)^ (FINDRISC, Peru) refer to a value ≥ 13 to detect DM2. Gabriel *et al*. (FINDRISC, Europe) ^11^, mentions an optimal cut-off point of ≥ 14 to identify persons with dysglycemia. Muñoz-González *et al*. (Venezuela) ^30^^)^ found that a score ≥ 14 points predicts diabetes mellitus or disorders of glucose metabolism and suggested that patients with LA-FINDRISC ≥ 14 points should have an oral glucose tolerance test ^30^. Makrilakis *et al*. ^16^, in Greece, (FINDRISC) mentioned a value ≥ 15 as the optimal point to predict DM2. Gomez-Arbelaez *et al*. ^25^^)^ in Colombia (FINDRISC), obtained an optimal cut-off point ≥ 14 points to identify DM2. Bernabe-Ortiz *et al*. ^28^^)^ in Peru (FINDRISC), showed that the optimal cut-off point was ≥ 11 to detect DM2 cases. In Spain (FINDRISC), Salinero-Fort *et al*. ^21^^)^ found that the best cutoff point was ≥ 13, based solely on the OGTT criteria.

For the different scales (FINDRISC, LA-FINDRISC and CUBDRISC), we found AUC-ROC values for persons with prediabetes of 0.831, 0.833 and 0.831, respectively, as well as 0.877, 0.880 and 0.877, respectively, for persons with DM2 (values considered of very good precision), which are higher than those described by other authors ^10^^-^^12^. This makes sense, since the questionnaire was applied to persons with at least one diabetes risk factor.

Likewise, when analyzing the study population according to sex, we found the optimal cutoff value ≥ 11 in men to predict dysglycemia with both the FINDRISC and LA-FINDRISC scale as well as with the CUBDRISC. However, in women, the optimal values for identifying persons with dysglycemia were ≥ 13 for the FINDRISC and ≥ 14 for the CUBDRISC; similar results have been found by other studies using the FINDRISC ^25^^,^^47^^,^^49^. In the case of the LA-FINDRISC scale for women the optimal value for detecting dysglycemia was also ≥ 11 similar to that of other studies ^27^^,^^29^^,^^30^.

We do not recommend using the LA-FINDRISC scale in Cuba because the waist circumference measurements are much higher ^26^^,^^29^^)^ than those used in Cuba ^35^, in addition to reducing the score for the risk of developing diabetes, as well as detecting fewer subjects with AFG and AGT than the CUBDRISC scale.

Our results suggest that a cutoff value ≥ 11 in men and ≥ 12 in women (cutoff point to identify prediabetes) is essential to indicate an OGTT in the search for both undiagnosed diabetes and prediabetes ^22^^,^^48^. In this study with the application of the FINDRISC and CUBDRISC scales, we found that persons with a score ≥ 13 are at higher risk for dysglycemia. Different validation studies of the FINDRISC scale showed similar cut-off points (between 12 and 14) to that of this study for the detection of dysglycemia ^11^^,^^12^^,^^25^.

The use of the FINDRISC and CUBDRISC risk scales allows us to define the population at higher risk of DM and, consequently, to intervene in a timely manner to eliminate or delay its onset. Efforts in this regard include raising awareness among primary care physicians, as well as the development of public policies for prevention and public education from an early age ^26^.

The cross-sectional design of our study is a limitation; therefore, our recommendation is to perform a follow-up of 10 years or more of the cohort to validate our risk predictions ^35^. Another limitation is that the participants came from an active screening, therefore, the results may not be extrapolated to the rest of the Cuban population. Most of the people were women, and this situation can be explained in part because there is greater compliance in attending medical appointments, they are more likely to participate in promotion and prevention programs and to complete questionnaires.

A third limitation has to do with the fact that the OGTT was not performed in persons who did not have risk factors for diabetes, so it was not possible to determine the sensitivity and specificity of the questionnaires, nor to calculate the ROC curves in these individuals. However, the diabetes screening in our study was not aimed at the general population, which emphasizes the application of the FINDRISC; LA-FINDRISC and CUBDRISC questionnaires in persons with diabetes risk factors.

It can be stated, therefore, that the FINDRISC questionnaire has been widely used, and that it has been proven in most cases that its use is beneficial for timely detection, providing also the benefit of low cost and ease of application ^10^^-^^12^^,^^21^^,^^27^^-^^29^^,^^43^. Therefore, the CUBDRISC questionnaire can also be considered an alternative for screening to identify persons with dysglycemia in the Cuban population.

The use of the FINDRISC and CUBDRISC risk scales is a simple, fast, non-invasive, reliable and inexpensive instrument that will allow the identification of individuals at risk of dysglycemia, which could be incorporated into the diabetes and family medicine program in Cuba, and would serve as a reference for other populations in the region with ethnic characteristics similar to ours. The use of these scales not only facilitates the detection of people with high glucose levels or unknown diabetes, but also makes it possible to exclude people from undergoing an OGTT.

We conclude that there is an almost perfect concordance between the scales. The CUBDRISC score proved to be a useful tool to identify people with dysglycemia in a Cuban population. It defined that people with score ≥ 13 have a higher risk of having some dysglycemia and those with score ≥ 12 and ≥ 14 have a higher risk of prediabetes and diabetes, respectively. However, other studies similar to this one are needed in other regions of Cuba.
