# Supplementary material for: FINDRISC modified for Cuba as a tool for the detection of prediabetes and undiagnosed diabetes in cuban population
Source: Rev Peru Med Exp Salud Publica. 2024 Dec 3;41(4):351–64. doi: 10.17843/rpmesp.2024.414.14138 (PMC11797583; doi:10.17843/rpmesp.2024.414.14138)
Supplement: Supplementary material. — Available in the electronic version of the RPMESP. [file rpmesp-41-04-14138-s001.docx]

Material suplementario

9056 adultos ≥ 20 años de 23 consultorios

Descartados por ausencia de factores de riesgo de diabetes (n = 905)

8151 adultos con al menos un factor de riesgo de diabetes

3737 personas analizadas sin diagnóstico previo de prediabetes o diabetes tipo 2

3909 individuos no analizados por razones logísticas imprevistas

505 excluidos con diagnóstico previo de diabetes

Figura. Diagrama de flujo que muestra la distribución de los sujetos.
