# Supplementary material for: FINDRISC modified for Cuba as a tool for the detection of prediabetes and undiagnosed diabetes in cuban population
Source: Rev Peru Med Exp Salud Publica. 2024 Dec 3;41(4):351–64. doi: 10.17843/rpmesp.2024.414.14138 (PMC11797583; doi:10.17843/rpmesp.2024.414.14138)
Supplement: Translation

# Translation: FINDRISC modificado para Cuba como herramienta para la detección de prediabetes y diabetes no diagnosticada en población cubana

**Authors:** Eduardo Cabrera-Rode, Oscar Díaz-Díaz, Neraldo Orlandi González, Mohan Ronald

**Affiliations:** 1 Instituto de Endocrinología, Universidad de Ciencias Médicas de La Habana, La Habana, Cuba.

**Keywords:** FINDRISC, LA-FINDRISC, Predicción, Cribado, Diabetes *Mellitus* tipo 2, Prediabetes, Disglucemia

## Abstract

**Objetivos.:**

Evaluar el Finnish Diabetes Risk Score (FINDRISC) modificado para Cuba como herramienta para la detección de prediabetes y diabetes no diagnosticada en una población cubana.

**Materiales y métodos.:**

Se realizó un estudio epidemiológico de corte transversal analítico y de fuente secundaria en 3737 adultos de 19 a mas años con al menos un factor de riesgo de diabetes, sin diagnóstico previo de prediabetes y diabetes *mellitus*, a los cuales se les realizó la prueba de tolerancia a la glucosa oral para el diagnóstico de disglucemia. Las puntuaciones de riesgo de diabetes aplicadas fueron la FINDRISC y las FINDRISC modificadas para Latinoamérica (LA-FINDRISC) y Cuba (CUBDRISC) con parámetros antropométricos propios. La curva ROC se utilizó para establecer el punto de corte de cada escala para el diagnóstico de disglucemia. Se calculó sensibilidad, especificidad, valores predictivos y razones de verosimilitud. La concordancia entre las escalas fue calculada con el coeficiente Kappa de Cohen.

**Resultados.:**

El 34,5% (n=1289) de las personas se diagnosticó con disglucemia (28,1% prediabetes y 6,4% diabetes tipo 2 sin diagnóstico previo). Las escalas LA-FINDRISC y CUBDRISC mostraron un grado de acuerdo casi perfecto con la escala FINDRISC para los distintos valores de corte del 11 al 16 (0,882-0,890 y 0,910-0,922, respectivamente). El punto de corte óptimo para detectar personas con disglucemia fue ≥ 13 para las escalas FINDRISC y CUBDRISC (sensibilidad 63,6% y 61,6%; especificidad 84,3% y 86,0%, respectivamente) y ≥11 para LA-FINDRISC (sensibilidad 58,0% y especificidad 88,0%).

**Conclusiones.:**

Existe una concordancia casi perfecta entre las escalas de riesgo de diabetes. El puntaje FINDRISC modificado para Cuba demostró ser una herramienta útil para identificar personas con prediabetes y diabetes con punto de corte 13 en una población cubana.

## INTRODUCCIÓN

La diabetes *mellitus* (DM) es un síndrome heterogéneo que se caracteriza por hiperglucemia crónica y trastornos en el metabolismo de la glucosa, los lípidos y las proteínas, como consecuencia de un déficit absoluto o relativo de secreción de insulina o resistencia a esta ^1^^,^^2^.

La prevalencia de DM ha aumentado considerablemente en el mundo ^(^^3^, En Centroamérica y Sudamérica se estima que alcanza una prevalencia de 9,5% ^3^. En el anuario estadístico del Ministerio de Salud Pública de Cuba del 2021, se reportó una tasa de prevalencia de DM de 66,9 por 1000 habitantes, tres veces mayor a la que existía hace 20 años ^(^^4^.

La diabetes no diagnosticada y la prediabetes son una gran preocupación por el alto riesgo de desarrollar complicaciones crónicas y el consiguiente aumento de los costos relacionados con estas. El atlas de la Federación Internacional de Diabetes (IDF, siglas en inglés) en su décima edición del 2021 señala que en la población mundial de 20 a 79 años, 537 millones de adultos padecen de DM (10,5%), 240 millones con DM no diagnosticada (44,7%) de los sujetos con DM, 541 millones presentan tolerancia a la glucosa alterada (TGA) (10,6%) y 319 millones muestran glucosa en ayunas alterada (GAA) (6,2%), con riesgo a desarrollar diabetes ^5^. En la región de Centroamérica y el Caribe un 32,8% de la DM no está diagnosticada ^5^. En Cuba, según la III Encuesta Nacional de Factores de Riesgo, el 42,0% de la población tenía una diabetes no conocida ^6^.

El interés por la prediabetes se ha incrementado en los últimos años, por su trascendencia como estado metabólico y condición predisponente para la progresión futura a la DM y enfermedad cardiovascular aterosclerótica, además de que conlleva una alta probabilidad de desarrollar muchas de las patologías normalmente asociadas a esa enfermedad, tales como retinopatía diabética, neuropatía periférica, enfermedad renal diabética y complicaciones macrovasculares ^2^^,^^7^^,^^8^.

En la actualidad, no existe consenso a nivel mundial sobre la estrategia para la detección de la DM y prediabetes. La glucemia en ayunas es la prueba más comúnmente utilizada para este fin, sin embargo, la prueba de tolerancia a la glucosa oral (PTGO) es el examen más específico para el diagnóstico de DM y prediabetes. La hemoglobina glucosilada (HbA1c) también es recomendada por algunos comités de expertos para el diagnóstico de esta enfermedad ^3^^,^^7^.

Para la identificación de sujetos con riesgo de diabetes tipo 2, existen cuestionarios simples, rápidos, económicos y no invasivos de puntuación, con los que se podría reducir el número de personas que tienen que someterse a una PTGO ^7^^,^^9^^-^^11^.

El cribado de la diabetes *mellitus* tipo 2 (DM2) y de prediabetes, con las intervenciones adecuadas, parece ser rentable ^9^. La Finnish Diabetes Risk Score (FINDRISC) es una de las herramientas de cribado más eficaz y utilizado para detectar nuevos casos de DM2 y prediabetes ^(^^10^^-^^12^. Sin embargo, FINDRISC debe validarse en poblaciones distintas de la población finlandesa original, con el fin de determinar los atributos de rendimiento (sensibilidad y especificidad) ^13^.

FINDRISC está disponible en casi todas las lenguas europeas (incluyendo el español), se ha validado en la mayoría de las poblaciones europeas ^10^^,^^11^^,^^14^^-^^21^^)^ y entre los países de lengua española ^22^ que ha sido validado se encuentran Colombia ^24^^-^^26^, Perú ^27^, Venezuela ^28^^,^^29^, México ^30^^,^^31^, España ^16^^,^^20^ y recientemente en Argentina ^12^.

En Latinoamérica, la escala FINDRISC ha sido modificada y utilizada en diferentes países, llamándose LA-FINDRISC ^26^^,^^28^^,^^29^. La primera vez se utilizó en una población colombiana, sobre la base de que la circunferencia cintura es mayor en esta población y que la obesidad abdominal es considerada factor de riesgo de diabetes, el valor de esta variable fue la única que se modificó en el FINDRISC, definiéndose como punto de corte ≥ 90 en la mujer y ≥ 94 en el hombre ^13^^,^^26^. Los resultados de las validaciones de los cuestionarios FINDRISC y LA-FINDRISC mostraron que existen diferentes valores de corte del puntaje con el que se puede predecir el riesgo de disglucemia, en dependencia del contexto de los países estudiados. El punto de corte para identificar personas con disglucemias en Europa oscilo entre 9 y 15 puntos ^(^^11^^,^^14^^-^^18^^,^^20^^,^^32^, mientras que en Latinoamérica fluctúo entre 9 y 14 puntos ^(^^12^^,^^24^^-^^26^^,^^29^^-^^31^.

En la bibliografía revisada se encontró que en Cuba se ha aplicado la escala FINDRISC en muestras pequeñas de la población de Cienfuegos y de Pinar del Río pero sin definir el valor de corte óptimo para identificar personas con disglucemias ^33^^,^^34^. En estas dos publicaciones anteriormente mencionadas se analiza únicamente la distribución de los diferentes puntajes de riesgo de diabetes en cada población.

En esta investigación se ha empleado una versión modificada de la escala FINDRISC para Cuba (CUBDRISC: por las siglas de CUBan Diabetes RIsk SCore) en la que se ha reemplazado los valores de la circunferencia de la cintura de obesidad abdominal para Europa por el punto de corte para cubanos ^35^. Como no hay estudios previos en Cuba que hayan evaluado la utilidad de las escalas LA-FINDRISC y CUBDRISC como herramientas de cribado de los trastornos de la regulación de la glucosa, se decidió realizar la presente investigación, cuyo objetivo fue evaluar el FINDRISC modificado para Cuba (CUBDRISC) como herramienta para la detección de personas con disglucemia (diabetes no diagnosticada y prediabetes) en una población cubana y comparar el rendimiento de esta puntuación con las escalas FINDRISC y LA-FINDRISC.

**MENSAJE CLAVE**

Motivación para el estudio. Existe un incremento de obesidad y diabetes mellitus en Cuba, por lo que es necesario aportar herramientas fáciles de emplear, rápidas y económicas para la identificación de personas con disglucemia.

Principales hallazgos. Por primera vez se establece en CUBA el punto de corte óptimo para los cuestionarios FINDRISC, LA-FINDRISC y FINDRISC modificado para Cuba (CUBDRISC) con parámetros antropométricos propios para identificar a las personas con disglucemia.

Implicaciones. El empleo de la escala CUBDRISC como herramienta simple, rápida y de bajo costo para la pesquisa activa de personas con disglucemia en población cubana y, en consecuencia, servirá para establecer estrategias de intervenciones oportunas a las personas con puntuación de riesgo para desarrollar disglucemia.

### MATERIAL Y MÉTODOS

### Población y diseño de estudio

El presente es un estudio epidemiológico, de corte transversal, analítico y de fuente secundaria (base de datos) en personas con al menos un factor de riesgo de diabetes ^2^^,^^7^, a partir de la base de datos de un estudio de pesquisa de disglucemia en población general realizada en el municipio Jaruco en 2008-2012 ^36^. Jaruco es un municipio de la provincia de Mayabeque en Cuba, situado a unos 30 km al este de La Habana. Tiene una extensión territorial de 275,7 km². Además del pueblo de Jaruco (aproximadamente 9,000 habitantes), el municipio incluye los poblados urbanos de San Antonio de Río Blanco, Caraballo y Bainoa.

Se entrevistaron 9056 personas adultas con edades ≥ 20 años (media: 53,0 y DE: 16,5) que provienen de 23 consultorios del pueblo de Jaruco, se descartó a 905 individuos por ausencia de factores de riesgo de diabetes, se identificaron 8151 adultos con al menos un factor de riesgo de diabetes ^2^^,^^7^, de estos 505 presentaban diabetes conocida, los cuales fueron excluidos. No se examinó a 3909 de los individuos por razones logísticas imprevistas y finalmente se analizaron a 3737 personas sin diagnóstico previo de prediabetes o diabetes tipo 2.

Se incluyeron las personas con al menos uno de los siguientes factores de riesgo de diabetes ^2^: Edad ≥ 45 años, adultos con sobrepeso u obesidad (IMC ≥ 25 kg/m^2^), antecedente familiar de diabetes en primero y segundo grado, circunferencia de la cintura ≥ 80 cm en mujeres y ≥ 90 cm en hombres, antecedente obstétrico de diabetes gestacional o hijos con peso > 4 kg al nacimiento, enfermedad isquémica coronaria o vascular de origen ateroesclerótico, hipertensión arterial, triglicéridos ≥ 150 mg/dL (1,7 mmol/l), colesterol HDL < 40 mg/dL (1,03 mmol/L), bajo peso al nacer o macrosomía, sedentarismo (< 150 minutos de actividad física/semana), síndrome de ovario poliquístico y Acantosis *nigricans*.

Se excluyeron las personas que padecían una enfermedad aguda, incapacidad mental evidente para dar información fidedigna, embarazadas, con diabetes o en tratamiento con medicamentos que modifiquen la glucemia. Además de cualquiera de las enfermedades endocrinas que se asocian con diabetes (síndrome de Cushing, hipertiroidismo, feocromocitoma, glucagonoma, acromegalia) o sospecha de insulinoma

### Definiciones

A todos los participantes se realizaron la PTGO, definido como método estándar de oro para el diagnóstico de prediabetes y de DM2 ^2^^,^^7^^,^^8^.

Para la clasificación se utilizaron los criterios diagnósticos de DM2 y de prediabetes, glucosa en ayunas alterada (GAA) y tolerancia a la glucosa alterada (TGA), de la guía cubana 2012, de la ALAD 2019 y de la ADA de 2024 ^2^^,^^7^^,^^8^, definiendo GAA con valores de glucemia en ayunas entre ≥ 5,6 mmol/L (100 mg/dL) y < 7 mmol/L (126 mg/dL) y a las 2 horas < 7,8 mmol/L (140 mg/dL). TGA con valores de glucemia en ayunas < 5,6 mmol/l (100 mg/dl) y a las 2 horas ≥ 7,8 (140 mg/dl) y < 11,1 mmol/L (200 mg/dL). Prediabetes mixta o doble con valores de glucemia en ayunas ≥ 5,6 mmol/L (100 mg/dL) y < 7 mmol/L (126 mg/dL) y a las 2 horas ≥ 7,8 (140 mg/dL) y < 11,1 mmol/L (200 mg/dL) y DM2 valores de glucemia en ayunas ≥ 7,0 mmol/L (126 mg/dL) y/o a las 2 horas ≥ 11,1 mmol/L (200 mg/dL) ^2^^,^^7^^,^^8^.

El término de «prediabetes» incluye la presencia de una glucemia de ayuna alterada (GAA), de una tolerancia a la glucosa alterada (TGA) o de ambas condiciones a la vez (GAA + TGA) ^7^^,^^8^, situaciones todas ellas que implican un riesgo elevado de desarrollar DM2 y de sufrir complicaciones cardiovasculares.

Para este estudio se consideró el término «disglucemia», a los estados metabólicos que cumplan los criterios de diabetes, de algún tipo de prediabetes o diabetes no diagnosticada previamente ^11^.

### Variables de estudio

A los sujetos se les midió el peso, talla, circunferencia de la cintura (CC) y se calculó el índice de masa corporal (IMC). La medida de la CC se tomó con una cinta métrica con el sujeto colocado de pie, en espiración, con el abdomen relajado, tomando como referencia el punto medio entre el borde inferior de la última costilla y la espina ilíaca anterosuperior de cada lado. En los casos de abdómenes péndulos la medición se realizó en el punto más prominente del abdomen.

Se realizó la medición de la tensión arterial (TA) a cada sujeto con esfigmomanómetro con mango de acorde al tamaño del brazo. Previamente, el sujeto estuvo sentado en reposo durante diez minutos. El procedimiento se realizó tres veces en el brazo derecho del sujeto, con un intervalo de cinco minutos. El valor final de la TA correspondió al promedio de las tres mediciones obtenidas.

A cada individuo se le realizó las pruebas de laboratorio en el momento de la primera extracción sanguínea (basal), luego de aproximadamente 8-12 horas de ayuno. A cada persona se le indicó la prueba de tolerancia a la glucosa oral (PTGO), con mediciones de glucemia (plasma venoso) en ayunas y a las dos horas después de una sobrecarga oral con 75,0 g de glucosa anhidra. Se utilizaron los siguientes datos de laboratorio: valores de glucosa en ayunas y a las dos horas luego de realizada la PTGO, así como colesterol y triglicéridos.

La información se extrajo de una base de datos proveniente del estudio epidemiológico Jaruco, la cual fue creada para la pesquisa activa de DM2 en la población de esa localidad y confeccionada por uno de los coautores ^(^^36^. Además, se obtuvieron las variables de edad, sexo, medidas antropométricas (peso, talla, IMC y CC) y TA o medicamentos para el tratamiento para hipertensión, así como también, los antecedentes familiares de DM (de primer y segundo grado), nivel elevado de glucemia conocido previamente reportado al momento de aplicado el cuestionario y consumo diario de vegetales y frutas. Asimismo, se recogió la actividad física realizada (150 minutos por semana).

### Cuestionario original FINDRISC

El FINDRISC es una sencilla herramienta de puntuación del riesgo de diabetes desarrollada originalmente en Finlandia para predecir la incidencia de diabetes entre la población finlandesa de 35 a 64 años ^10^. Se basa en ocho factores sencillos de riesgo de diabetes, como la edad (años), el IMC (kg/m^2^), la circunferencia de la cintura (cm), los antecedentes de hipertensión arterial, los antecedentes de aumento de la glucemia, los antecedentes familiares de diabetes, el consumo diario de frutas o verduras y la actividad física diaria ^10^. La herramienta no requiere pruebas de laboratorio y tiene diferentes puntuaciones ponderadas en función del riesgo asociado, con una puntuación final que oscila entre 0 y 26 ^10^.

El nivel de riesgo para diabetes se evalúa en cinco categorías ^10^: menor a 7 puntos (bajo riesgo, 1% desarrollará DM2); entre 7-11 puntos (ligeramente elevado, 4% desarrollará DM2); entre 12-14 puntos (moderado riesgo, 17% desarrollará DM2); entre 15-19 puntos (alto riesgo, 33% desarrollará DM2); mayor a 20 puntos (muy alto riesgo, 50% desarrollará DM2).

Con los datos de las variables anteriormente mencionadas para cada individuo se empleó el cuestionario FINDRISC. Las variables y sus puntajes se describen en la tabla 1.

**Tabla 1:** Variables de los cuestionarios FINDRISC ^10^ y de los modificados para Latinoamérica (LA-FINDRISC) ^30^ y para Cuba (CUBDRISC) ^36^ con sus respectivos puntajes.

				Puntos
1) Edad				
	Menos de 45 años			0 punto
	45 a 54 años			2 puntos
	55 a 64 años			3 puntos
	Más de 64 años			4 puntos
2) Índice de masa corporal				
	Menos de 25 Kg\m^2^			0 punto
	25 - 30 Kg\m^2^			1 punto
	Mayor de 30 Kg\m^2^			3 puntos
3) Circunferencia de la cintura				
	FINDRISC ^10^	LA-FINDRISC ^30^	CUBDRISC ^36^	
	H: menor 94 cm	H: menor 94 cm	H: menor 90 cm	0 punto
	M: menor 80 cm	M: menor 90 cm	M: menor 80 cm	
	H: 94-102 cm	-	-	3 puntos
	M: 80-88 cm	-	-	
	H: más de 102 cm	H: ≥94 cm	H: ≥90 cm	4 puntos
	M: más de 88 cm	M: ≥90 cm	M: ≥80 cm	
4) ¿Usualmente realiza actividad física durante 30 minutos por día?				
	Sí			0 punto
	No			2 puntos
5) ¿Con que frecuencia usted consume vegetales o Frutas?				
	A diario			0 punto
	No a diario			1 punto
6) ¿Alguna vez ha tomado medicamentos antihipertensivos de forma regular?				
	No			0 punto
	Sí			2 puntos
7) ¿Alguna vez ha presentado cifras elevadas de glucemia (durante un chequeo, embarazo o algún día que estuvo enfermo)?				
	No			0 punto
	Sí			5 puntos
8) Algún miembro de su familia ha sido diagnosticado con diabetes tipo 1 o tipo 2				
	No			0 punto
	Sí (abuelos, tíos, primos, sobrinos, mujer con DG)			3 puntos
	Sí (padres, hermanos, hijos)			5 puntos
El riesgo de desarrollar una diabetes mellitus en 10 años es:				
	Puntuación total:	Riesgo de desarrollar diabetes:	Interpretación nivel de riesgo:	
	Menos de 7 puntos	1%	Bajo	
	De 7-11 puntos	4%	Ligeramente elevado	
	De 12-14 puntos	17%	Moderado	
	De 15-20 puntos	33%	Alto	
	Más 20 puntos	50%	Muy alto	

H: hombres, M: mujeres, DG: diabetes gestacional

### FINDRISC modificados para Latinoamérica y Cuba

Los cuestionarios modificados LA-FINDRISC ^28^^,^^29^ y CUBDRISC también se usaron para cada persona y constan igualmente de ocho variables: edad, IMC, CC, actividad física, consumo diario de verduras y frutas, uso de fármacos antihipertensivos, antecedentes personales de hiperglucemia y antecedentes familiares de diabetes. Sin embargo, los puntos de corte de la CC se ajustaron para América Latina (CC ≥ 94 cm para los hombres y ≥ 90 cm para las mujeres) ^(^^13^^,^^29^ y para Cuba (CC ≥ 90 cm para los hombres y ≥ 80 cm para las mujeres) ^(^^35^, añadiendo 4 puntos para los sujetos con obesidad abdominal y ningún punto para aquellas personas con valores inferiores de los puntos de corte de la CC ^28^^,^^29^, la puntuación total oscila entre 0 y 26 puntos. Ver escalas modificadas de riesgo de desarrollar una DM2 en 10 años (Tabla 1).

### Análisis estadístico

En el análisis descriptivo, las variables cualitativas incluidas en los cuestionarios empleados [edad, IMC e circunferencia de la cintura categorizadas, práctica o no de actividad física, consumo diario o no de vegetales y frutas, tratamiento o no para la HTA, antecedentes o no de hiperglucemia e historia o no de familiares de diabetes o diabetes gestacional), con la presencia (o no) de disglucemias en general, prediabetes y diabetes en particular] se expresaron en frecuencia absoluta y sus respectivos porcentajes (%). Se utilizó la prueba de Chi cuadrado para comparación de proporciones.

La capacidad predictiva y el desempeño de las escalas FINDRISC, LA-FINDRISC y CUBDRISC en la detección de disglucemia (DM2 no conocida y prediabetes), se determinó mediante el cálculo de la sensibilidad, la especificidad, valor predictivo positivo (VPP), valor predictivo negativo (VPN) y el área bajo la curva de características operativas del receptor (AUC-ROC). Se consideró que la variable tendrá un buen poder discriminatorio cuando el área bajo la curva ROC sea diferente de 0,5 (p < 0,05 e intervalo de confianza que no contenga al 0,5). Una prueba diagnóstica perfecta tiene un AUC de 1,0. La precisión de una prueba depende del AUC-ROC (AUC 0,9-1,0 excelente; 0,8-0,9 muy bueno; 0,7-0,8 bueno; 0,6-0,7 suficiente; 0,5-0,6 malo; <0,5 prueba no útil) ^(^^37^.

Se calcularon los puntos de corte para cada cuestionario desde la puntuación 11 hasta la 16. Se calculó también las razones de verosimilitud (likelihood ratio) para determinar la utilidad clínica de la prueba diagnóstica para los distintos puntos de cortes de las escalas utilizadas (FINDRISC, LA-FINDRISC y CUBDRISC). Se calculó los respectivos intervalos de confianza al 95% (IC95%). Se analizó la capacidad predictiva de cada cuestionario al comparar personas no disglucemicas contra disglucemicos (prediabetes y diabetes detectada) y por sexo, así como entre prediabetes versus personas con tolerancia a glucosa normal y diabetes contra personas con tolerancia a glucosa normal.

Se utilizó la razón de verosimilitud (LR) para identificar la puntuación con la mejor capacidad discriminatoria basada en estimaciones de AUC-ROC, especificidad y sensibilidad ^38^. Una razón de verosimilitud positivo (LR+) > 2 y una razón de verosimilitud negativo < 0,5 se consideró de útil.

El grado de concordancia diagnóstica de los valores de cortes obtenidos entre las escalas modificadas (LA-FINDRISC y CUBDRISC) con la escala original FINDRISC (patrón de oro) se calculó por el coeficiente kappa de Cohen. La interpretación del coeficiente kappa se realizó con base en los seis niveles de fuerza de concordancia propuestos por Landis y Koch ^39^: ≤ 0.00 (pobre); 0.01-0.20 (leve); 0.21-0.40 (aceptable), 0.41-0.60 (moderada); 0.61-0.80 (considerable) y 0.81-1.00 (casi perfecta). Se admitió como valores de significación estadística los valores de p menores que 0,05.

Para el análisis de los datos se utilizó el programa estadístico SPSS para Windows® versión 19.0 y Epidat versión 3.1. Se adoptó un nivel de significancia α = 0.05.

### Aspectos éticos

Para esta investigación se usó la base de datos del estudio Jaruco, pero se conservó la confidencialidad de las personas que se estudiaron y para el análisis de los datos se contó con la aprobación del comité de ética de investigación (CEI) del Instituto de Endocrinología (INEN) (Código: I070LH2304, 7 de octubre de 2021). El estudio cuenta con la autorización del investigador principal del estudio original. Los investigadores participantes asumen el compromiso de honestidad en los análisis y el reporte de los resultados.

## RESULTADOS

### Características demográficas, antropométricas, clínicas y bioquímicas de los participantes

Se estudiaron 3737 personas (material suplementario) con algún factor de riesgo de diabetes mellitus de ellos predominaron los mayores de 45 años (65,3%) sobre los menores de 45 años (34,7%). Sin embargo, al distribuir por diferentes grupos etarios encontramos diferencias entre hombres y mujeres (p=0,003; Tabla 2). Predominó el sexo femenino sobre el masculino (Tabla 3). Existió un predominio de personas de piel blanca seguida de los que presentan piel mestiza y negra, respectivamente. Según el IMC, el 35,9% estaba en sobrepeso y el 17,6% mostró obesidad; respectivamente, además presentaron circunferencia abdominal alterada el 67,6% de los individuos y el 47,5% de los sujetos tenían HTA o tratamiento. Las mujeres presentaron mayor proporción de obesidad, obesidad abdominal e HTA que los hombres (Tabla 2).

**Tabla 2:** Características demográficas, antropométricas, clínicas y bioquímicas en las personas con al menos un factor de riesgo de diabetes *mellitus* tipo 2.

Características antropométricas clínicas y bioquímicas	Total n (%) n=3737	Mujeres n (%) n=2194	Hombres n (%) n=1543
Edad			
20 - 39 años	866 (23,2)	542 (24,7)	324 (21,0)
40 - 59 años	1545 (41,3)	923 (42,1)	622 (40,3)
60 - 79 años	1086 (29,1)	595 (27,1)	491 (31,8) ^a^
≥ 80 años	240 (6,4)	134 (6,1)	106 (6,9)
Color de piel			
Blanca	2970 (79,5)	1375 (79,1)	1235 (80,0)
Negra	363 (9,7)	219 (10,0)	144 (9,3)
Mestiza	404 (10,8)	240 (10,9)	164 (10,6)
Índice de masa corporal			
Normopeso o bajo peso	1737 (46,5)	948 (43,2)	789 (51,1)
Sobrepeso	1341 (35,9)	792 (36,1)	549 (35,6)
Obesidad	659 (17,6)	454 (20,7) ^b^	205 (13,3)
Circunferencia de cintura (H ≥ 90 cm y M ≥ 80 cm)	2528 (67,6)	1660 (75,7) ^b^	868 (56,3)
Hipertensión arterial (≥130/85 mmHg o tratamiento)	1774 (47,5)	1037 (52,7) ^b^	737 (47,8)
Disglucemia			
Diabetes detectada	238 (6,4)	135 (6,2)	103 (6,7)
GAA	798 (21,4)	425 (19,4)	373 (24,2) ^c^
TGA	132 (3,5)	81 (3,7)	51 (3,3)
GAA + TGA	121 (3,2)	65 (3,0)	56 (3,6)
No disglucemia	2448 (65,5)	1488 (67,8)	960 (62,2)
Colesterol ≥ 5,2 mmol/L (200 mg/dL)	978^e^ (26,2)	635 ^g^ (28,9) ^b^	343 ^h^ (22,2)
Triglicéridos ≥ 1,7 mmol/L (150 mg/dL)	1050 ^f^ (28,1)	565 ^g^ (25,8)	485 ^i^ (31,4) ^d^

a p= 0,003 entre grupos etareos por sexos, ^b^ p<0,0001 vs masculino ^c^ p=0,0005 vs femenino ^d^ p=0,0002 vs femenino

e n=3732, ^f^ n=3734, ^g^ n=2193, ^h^ n=1539, ^i^ n=1541. Se usa la prueba de Chi cuadrado para todas las variables.

GAA: glucosa en ayunas alterada, TGA: tolerancia a la glucosa alterada, GAA + TGA: ambas condiciones a la vez, H: hombres, M: mujeres.

En relación con el nivel de educación, el 8,8% de los adultos tenia nivel universitario (329/3737), el 17,8% (667/3737) preuniversitario, el 12,9% (483/3737) técnico medio y el 38,5% con secundaria (1438/3737).

Se identificaron con prediabetes (GAA, TGA o GAA/TGA) el 28,1% y el 6,4% de las personas con DM2 detectada. Se observó que los hombres mostraron mayor frecuencia de GAA que las mujeres (p=0,0005) (Tabla 2). En el patrón lipídico se encontró, que el 28,1% de los individuos tenían hipertriglicéridemia y un 26,2% poseía hipercolesterolemia. Las mayores frecuencias de hipercolesterolemia e hipertrigliceridemia fueron en las mujeres y hombres, respectivamente (Tabla 2).

### Frecuencias de las variables del cuestionario original (FINDRISC)

En la muestra de estudio, el 34,7% de las personas tenía menos de 45 años y en el resto de los grupos de edades la frecuencia disminuyó discretamente (Tabla 3). Según la circunferencia de cintura, se encontró un 38,0% de los sujetos sin obesidad abdominal; un 29,9% con obesidad abdominal leve y el 32,1% con obesidad abdominal severa (Tabla 3).

**Tabla 3:** Frecuencias de las variables del cuestionario original (FINDRISC) en las personas con al menos un factor de riesgo de diabetes mellitus tipo 2.

Variable		n (%)
Edad (años)		
	20-44	1295 (34,7)
	45-54	744 (19,9)
	55-64	722 (19,3)
	≥65	976 (26,1)
Sexo		
	Femenino	2194 (58,7)
	Masculino	1543 (41,3)
Índice de masa corporal		
	Bajo peso o normo peso	1737 (46,5)
	Sobrepeso	1341 (35,9)
	Obesos	659 (17,6)
Circunferencia de cintura (cm)		
	Sin obesidad abdominal (H <94 y M <80)	1421 (38,0)
	Obesidad abdominal leve (H 94-102 y M 80-88)	1116 (29,9)
	Obesidad abdominal severa (H >102 y M >88)	1200 (32,1)
Actividad física ^a^		
	Sí	1990 (53,3)
	No	1747 (46,7)
Consume vegetales y frutas ^b^		
	Sí	649 (17,4)
	No	3088 (82,6)
Ha tenido HTA o tratamiento		
	Sí	1774 (47,5)
	No	1963 (52,5)
Antecedentes de glucemia alta		
	Sí	1326 (35,5)
	No	2411 (64,5)
Antecedentes de familiares de primer grado con diabetes o de diabetes gestacional		
	Sí	1116 (29,9)
	No	2621 (70,1)

H: hombres, M: mujeres

a Realiza actividad física al menos 30 minutos al día, ^b^ consume a diario vegetales y/o frutas

Se constató que el 53,3% de las personas realizaban actividad física y el 17,4% consumía vegetales y frutas diariamente. El 47,5% de los evaluados eran hipertensos o tomaron antihipertensivos. Además, el 29,9% tenía antecedente familiar de primer grado con diabetes o diabetes gestacional. Finalmente, el 35,5% presentó alguna vez cifras elevadas de glucemia (Tabla 3).

### Concordancia diagnóstica de los diversos puntos de cortes según los cuestionarios estudiados

Las escalas modificadas LA-FINDRISC y CUBDRISC mostraron un grado de acuerdo casi perfecto con la escala original FINDRISC para los distintos valores de corte del 11 al 16 (0,882-0,890 y 0,910-0,922, respectivamente) (Tabla 4).

**Tabla 4:** Concordancia diagnóstica de los diversos puntos de cortes de la escala original FINDRISC con las escalas modificadas (LA-FINDRISC y CUBDRISC).

	Escalas modificadas	Kappa	Error estándar	Significancia	Grado de concordancia
FINDRISC ≥ 11 p	LA-FINDRISC	0,882	0,008	<0,0001	Casi perfecta
	CUBDRISC	0,910	0,007	<0,0001	Casi perfecta
FINDRISC ≥ 12 p	LA-FINDRISC	0,893	0,007	<0,0001	Casi perfecta
	CUBDRISC	0,915	0,007	<0,0001	Casi perfecta
FINDRISC ≥ 13 p	LA-FINDRISC	0,904	0,007	<0,0001	Casi perfecta
	CUBDRISC	0,921	0,006	<0,0001	Casi perfecta
FINDRISC ≥ 14 p	LA-FINDRISC	0,907	0,007	<0,0001	Casi perfecta
	CUBDRISC	0,933	0,006	<0,0001	Casi perfecta
FINDRISC ≥ 15 p	LA-FINDRISC	0,888	0,009	<0,0001	Casi perfecta
	CUBDRISC	0,924	0,007	<0,0001	Casi perfecta
FINDRISC ≥ 16 p	LA-FINDRISC	0,890	0,009	<0,0001	Casi perfecta
	CUBDRISC	0,922	0,007	<0,0001	Casi perfecta

Concordancia diagnóstica: Coeficiente kappa ≤ 0.00 (pobre); 0.01-0.20 (leve); 0.21-0.40 (aceptable), 0.41-0.60 (moderada); 0.61-0.80 (considerable) y 0.81-1.00 (casi perfecta).

### Puntos de corte para identificar personas con disglucemias según los cuestionarios analizados

La capacidad de los cuestionarios FINDRISC y CUBDRISC para identificar personas con disglucemias, el punto de corte óptimo fue de ≥ 13. En cambio, para la escala LA-FINDRISC el punto de corte óptimo fue de ≥ 11 para detectar personas con disglucemias (Tabla 5).

**Tabla 5:** Comparación de personas no diglucémicos contra disglucemicos (prediabetes y diabetes detectada) según varios puntos de corte de los cuestionarios analizados.

Escala	Puntos de corte	S IC95%	E IC95%	VPP IC95%	VPN IC95%	LR+ IC95%	LR- IC95%	AUC IC95%	Valor de p
FINDRISC	≥ 11	55,7 53,5-57,9	89,0 87,6-90,5	85,0 83,0-87,0	64,4 62,5-66,3	5,1 4,4-5,8	0,50 0,47-0,52	0,745 0,733-0,760	
LA-FINDRISC		58,0 55,7-60,3	88,0 86,5-89,5	82,2 80,1-84,4	68,7 66,8-70,5	4,8 4,3-5,5	0,48 0,45-0,50	0,755 0,741-0,768	<0,0001
CUBDRISC		53,4 51,2-55,5	90,6 89,2-92,1	88,4 86,6-90,2	59,4 57,4-61,3	5,7 4,9-6,7	0,41 0,49-0,54	0,739 0,726-0,752	
FINDRISC	≥ 12	59,1 56,7-61,4	86,4 84,9-87,9	78,7 76,5-81,0	71,2 69,4-73,0	4,4 3,9-4,9	0,47 0,45-0,50	0,750 0,736-0,764	
LA-FINDRISC		60,7 58,3-63,2	84,9 83,4-86,4	74,8 72,4-77,2	74,6 72,8-76,3	4,0 3,6-4,5	0,46 0,43-0,49	0,747 0,732-0,761	<0,0001
CUBDRISC		56,3 54,1-58,6	87,6 86,1-89,1	82,1 80,0-84,2	66,5 64,6-68,4	4,5 4,0-5,2	0,50 0,47-0,53	0,743 0,729-0,757	
FINDRISC	≥ 13	63,6 61,1-66,1	84,3 82,8-85,8	72,3 69,8-74,8	78,2 76,5-79,8	4,0 3,7-4,5	0,43 0,40-0,46	0,753 0,738-0,767	
LA-FINDRISC		65,3 62,7-67,9	83,0 81,5-84,5	68,5 66,0-71,1	80,8 79,3-82,4	3,8 3,5-4,2	0,42 0,39-0,45	0,747 0,732-0,762	<0.0001
CUBDRISC		61,6 59,2-64,0	86,0 84,5-87,5	77,0 74,6-79,3	74,8 73,0-76,5	4,4 3,9-4,9	0,45 0,42-0,48	0,759 0,744-0,773	
FINDRISC	≥ 14	68,1 65,4-70,7	82,1 80,6-83,6	65,3 62,7-68,0	83.9 82.4-85.3	3,8 3,5-4,2	0.39 0.36-0.42	0,745 0,731-0.761	
LA-FINDRISC		69,2 66,5-72,0	80,6 79,1-82,2	60,9 58,2-63,6	85,7 84.3-87.2	3,6 3,3-3,9	0,38 0,35-0,42	0,733 0,718-0,748	<0,0001
CUBDRISC		66,4 63,8-68,9	83,5 82,0-85,0	69,5 67,0-72,1	81,5 80,0-83.0	4,0 3,7-4,5	0,40 0,37-0,43	0,755 0,740-0,770	
FINDRISC	≥ 15	72,2 69,5-75,0	80,0 78,5-81,5	58,1 55,4-60,8	88,2 86.9-89.5	3,6 3,3-3,9	0.35 0.31-0.38	0,732 0,717-0,747	
LA-FINDRISC		74,4 71,6-77,3	79,0 77,4-80,5	54,4 51,6-57,1	90.2 89.0-91.4	3,5 3,3-3,8	0,32 0.29-0.36	0,723 0,708-0,738	<0,0001
CUBDRISC		70,5 67,8-73,2	81,6 80,1-83,1	63,2 60,5-65,8	86.1 84.7-87.5	3,8 3,5-4,2	0,36 0,33-0,40	0,746 0,731-0,761	
FINDRISC	≥ 16	76,7 73,8-79,7	77,6 76,1-79,2	49,7 46,9-52,4	92.1 91.0-93.2	3,4 3,2-3,7	0,30 0.26-0.34	0.829 0.812-0.845	
LA-FINDRISC		79,2 76,3-82,2	76,9 75,3-78,4	46,5 43,7-49,2	93,4 92.6-94.6	3,4 3,2-3,7	0,27 0,23-0,31	0,700 0,686-0,715	<0,0001
CUBDRISC		75,2 72,4-78,0	79,2 77,7-80,7	54,9 52,1-57,6	90,5 89,3-91,7	3,6 3,4-3,9	0,31 0,28-0,35	0,727 0,712-0,741	

S: sensibilidad, E: especificidad, VPP: valor predictivo positivo, VPN: valor predictivo negativo. IC95%: intervalo de confianza al 95%, LR+: razón de verosimilitud positiva, LR-: razón de verosimilitud negativa, AUC: área bajo la curva.

Al analizar la frecuencia de disglucemias en los sujetos de acuerdo con la presencia o no del punto de corte óptimo ≥ 13 para las escalas FINDRISC y CUBDRISC, notamos que las personas con esta puntuación mostraron una mayor proporción de disglucemia (prediabetes o DM2) (63,6% y 61,6%, respectivamente) que aquellas con el puntaje menor de 13 (15,7 % y 13,9%, respectivamente) (p<0,0001). Sin embargo, en las personas, de acuerdo con la presencia o no del punto de corte ≥ 11 para la escala LA-FINDRISC, se observó que con esa puntuación tenían una mayor proporción de disglucemia (58,0%) que aquellas con el puntaje menor de 11 (12,0%) (p < 0,0001) (datos no mostrados).

### Área bajo la curva y puntos de corte óptimos para la detección de prediabetes o diabetes desconocida

La figura 1 muestra las curvas AUC-ROC separadas para la detección de diabetes no diagnosticada previamente y la prediabetes en la muestra analizada. El AUC para la prediabetes fue de 0,831 (IC95%: 0,817-0,845) para los cuestionarios FINDRISC y CUBDRISC, mientras que para la detección de diabetes fue de 0,877 (IC95%: 0,817-0,845) para FINDRISC y 0,877 (IC95%: 0,816-0,845) para la CUBDRISC. En cambio, para la escala LA-FINDRISC la identificación de prediabetes el AUC fue de 0,833 (IC95%: 0,819-0,847) y para diabetes fue de 0,880 (IC95%: 0,857-0,902).

**Figura 1:** Área bajo la curva (AUC) para la detección de prediabetes o diabetes desconocida en comparación con las personas sin disglucemias al aplicar las distintas escalas.

El punto de corte óptimo para las escalas FINDRISC y CUBDRISC para identificar prediabetes fue de ≥ 12 puntos y para diabetes fue de ≥ 13 y ≥ 14 puntos, respectivamente. En contraste para la escala LA-FINDRISC para prediabetes el mejor punto de corte fue de ≥ 11 puntos y para diabetes fue de ≥ 12 puntos (datos no mostrados).

### Puntos de corte óptimos para identificar personas con disglucemias de acuerdo con el sexo

El punto de corte óptimo para identificar personas con disglucemias en las escalas FINDRISC y CUBDRISC para hombres fue de ≥11 puntos (sensibilidad= 67,5% y 62,3%, especificidad= 86,3% y 89,0%, índice de verosimilitud positivo de 4,9 y 5,7, AUC de 0,783 y 0,772, respectivamente) y ≥13 puntos para mujeres (sensibilidad=57,9%, especificidad=88,4%, índice de verosimilitud positivo de 5,0 y AUC de 0,761) pronosticó disglucemia en las submuestras estudiadas empleando la escala FINDRISC. Por el contrario, la escala CUBDRISC con un punto de corte de ≥14 puntos identificó a las mujeres con disglucemia (sensibilidad= 61,6%, especificidad= 86,7%, índice de verosimilitud positivo de 4,6 y AUC 0,763). Al analizar los puntos de corte óptimos para hombres y mujeres para la LA-FINDRISC fue de 11 para ambos sexos (sensibilidad= 65,2% y 53,3%, especificidad= 86,7% y 89,0, índice de verosimilitud positivo de 4,9 y 4,8, AUC 0,775 y 0,742, respectivamente) (datos no mostrados).

## DISCUSIÓN

Las cifras elevadas en las prevalencias e incidencias de la DM2 han generado la necesidad de desarrollar herramientas de cribado para diagnosticar y predecir el riesgo de la enfermedad a nivel mundial ^3^^,^^5^. De todos los cuestionarios estudiados, el FINDRISC es posiblemente el más aceptado, y ha sido usado en los estudios de intervención poblacional principalmente en Europa ^10^^-^^12^^,^^15^^-^^23^^,^^33^, Latinoamérica ^24^^,^^25^^,^^27^^-^^30^^,^^32^^)^ y el modificado para Latinoamérica (LA-FINDRISC) ^26^^-^^30^.

La intervención hacia la DM2, por su elevada prevalencia y sus complicaciones tanto a corto como a largo plazo, puede lograrse mediante la prevención y predicción precoz de la disglucemia ^11^^,^^13^^,^^15^^,^^16^^,^^33^^,^^40^^-^^43^. Al igual que otros estudios de cribado poblacional, hemos escogido al cuestionario FINDRISC ^10^^-^^12^^,^^20^^-^^23^^,^^25^^,^^28^^-^^30^^)^ y LA-FINDRISC ^26^^-^^30^^)^ por la facilidad de obtener parámetros demográficos, antropométricos, clínicos y de antecedentes familiares para identificar a personas con disglucemias ^11^^,^^12^^,^^14^^-^^16^^,^^19^^,^^21^^,^^26^^,^^29^^-^^31^^,^^33^.

Este es el primer estudio adaptado a la población cubana en el que se calcula la puntuación del riesgo de diabetes y prediabetes para las escalas FINDRISC, LA-FINDRISC y la modificada para Cuba (CUBDRISC). Validar la escala CUBDRISC para la población cubana es una tarea importante debido a que las puntuaciones óptimas para cada uno de los elementos evaluados resultan variables para cada población ^13^.

En un análisis multivariado que incluía todos los parámetros de la cohorte original de FINDRISC, una edad > 45 años aumento el riesgo de DM2 ^10^. En nuestro estudio la mayoría de los sujetos tenían una edad de ≥ 45 años (n=2442), por lo que el riesgo asociado a la edad fue mayor.

Naranjo *et al*. ^35^ en su estudio incorporaron una alta proporción de individuos con sobrepeso y obesidad (80,6%). Al comparar la proporción antes mencionada con los individuos con exceso de peso analizados en esta investigación (54,5%), los mismos son superiores. En este estudio las mujeres mostraron mayor frecuencia de obesidad en comparación con los hombres (20,7% vs 13,3%, respectivamente). No obstante, la frecuencia de obesidad abdominal según las categorías del FINDRISC entre nuestra investigación y la de Naranjo *et al*. fueron similares al analizar ambos sexos ^35^. Sin embargo, las mujeres presentaron mayor proporción de obesidad abdominal (≥ 80 cm) ^36^ con relación a los hombres (≥ 90 cm) ^(^^36^ (75,7% y 56,3%, respectivamente).

La alta proporción de mujeres con HTA en este estudio debe ser el resultado del incremento de la frecuencia de obesidad en las mujeres en comparación con los hombres. Datos disímiles a los encontrados en la Encuesta Nacional de Factores de Riesgo donde no existió diferencias de la prevalencia de hipertensos por sexo ^6^.

El cuestionario FINDRISC incluye el consumo diario de frutas y vegetales, aunque esta variable contribuyo muy poco en el poder predictivo de la diabetes en el estudio FINDRISC ^10^. El hecho de que la mayor parte de los individuos no consumieran frutas y verduras a diario, subraya la importancia de abordar este factor en los programas de prevención de DM2. En un estudio anterior realizado en la atención primaria de Pinar del Río, se encontró también un bajo consumo diario de frutas y verduras.^35^ En cambio en varios estudios ^(^^10^^,^^11^^,^^25^, en los que se aplicó esta variable, el consumo diario de frutas y verduras fue superior al de este estudio y al de Naranjo *et al*. ^35^ Sin embargo fue similar a un estudio venezolano ^30^.

En esta investigación, obtuvimos 6,4% de individuos con DM2 detectada, así como un 28,1% de personas con prediabetes, utilizando la PTGO como prueba diagnóstica. En total, 1289 (34,5%) pacientes presentaron alguna de las dos condiciones antes mencionadas. La frecuencia de alteraciones del metabolismo de la glucosa en esta investigación fue un poco más alta a la encontrada en la atención primaria en un estudio multicéntrico en Europa (30,7%; 3512/11444) ^11^. Lo antes mencionado es razonable pues esta investigación se ejecutó en personas con al menos un factor de riesgo de diabetes ^2^^,^^7^^,^^8^.

Con relación a la frecuencia de prediabetes (TGA y GAA/TGA) y DM2 detectada, no obtuvimos diferencias en relación al sexo, excepto en la GAA, donde la misma prevaleció en el sexo masculino con respecto al femenino (24,2% vs 19,4%; respectivamente), esto es incongruente con la mayoría de los estudios realizados en Europa con mayor prevalencia de DM2, en los cuales predominó el sexo masculino ^10^^,^^11^. Por otra parte, la similar frecuencia de DM2 detectada en ambos sexos, no coincide con lo reportado en Cuba, donde la mayor prevalencia de DM2 ha sido en el sexo femenino ^4^.

La proporción de DM2 detectada (n=238) y prediabetes (1051) en esta investigación sugiere un aumento sustancial de prevalencia de DM2 en los próximos 10 años, si no se toman medidas preventivas eficaces. Observamos en esta investigación que el presentar un riesgo individual de moderado (12-14 puntos), alto (15-20 puntos) a muy alto (más de 20 puntos), se eleva la frecuencia de personas con disglucemia. Hay varios estudios que han hallado resultados similares ^12^^,^^17^^,^^44^.

En el análisis de los datos tomando en cuenta los distintos tipos de prediabetes y la DM2, observamos que las personas con prediabetes y DM2 tuvieron una puntuación ≥ 12 con las siguientes proporciones para las distintas escalas de riesgo de diabetes (FINDRISC, LA-FINDRISC y CUBDRISC): FINDRISC [GAA 74,9%; TGA 78,0%, prediabetes doble (GAA/TGA) 94,2% y DM2 del 84,0%]. LA-FINDRISC [GAA 70,6%; TGA 68,9%, prediabetes doble (GAA/TGA) 93,4% y DM2 del 82,8%] y en la CUBDRISC [GAA 78,6%; TGA, 82,6%, prediabetes doble (GAA/TGA) 95,9% y DM2 del 86,6%]. Los datos antes mencionados de las distintas escalas nos indican que estos cuestionarios detectan una gran cantidad de personas con disglucemia. Diversos estudios que emplearon las escalas FINDRISC y LA-FINDRISC han coincidido con nuestros resultados ^12^^,^^13^^,^^25^^,^^29^^,^^31^^,^^33^. Consecuentemente, la escala CUBDRISC detectó mayor frecuencia de personas con GAA y TGA que la escala LA-FINDRISC (p=0,0003 y 0,0146, respectivamente), lo cual es razonable, pues los valores de corte de la circunferencia de la cintura utilizados para la escala CUBDRISC ^36^ son más bajos que los empleados en las escalas FINDRISC ^10^ y LA-FINDRISC ^(^^29^^,^^30^^,^^45^.

Pocos estudios comparan simultáneamente más de dos escalas de riesgo de diabetes (FINDRISC, LA-FINDRISC y CUBDRISC). En otras poblaciones de Latinoamérica y de Europa se han encontrado resultados similares entre las distintas escalas de riesgo, hallándose concordancia entre ellas ^28^^,^^29^^,^^46^. Tales estudios han considerado válida la escala LA-FINDRISC ^28^^,^^29^. Esta investigación reveló un grado de concordancia casi perfecto según el coeficiente kappa entre las escalas LA-FINDRISC y CUBDRISC con la escala original FINDRISC para todos los puntos de cortes estudiados.

Los autores sugieren que la implementación del CUBDRISC y FINDRISC en población cubana sería una alternativa útil para detectar personas con disglucemia (prediabetes y diabetes) especialmente, en entornos con recursos limitados, como son aquellos en donde la glucemia en ayunas u otro marcador, no están disponibles ^28^. Resultados semejantes se obtuvieron con la prueba de FINDRISC y LA-FINDRISC que tuvieron un desempeño adecuado como herramienta para la detección de disglucemia en estudios transversales ^11^^,^^21^.

El FINDRISC se desarrolló originalmente en una cohorte prospectiva para identificar a las personas con alto riesgo de desarrollar DM2 ^10^, y los estudios transversales que han analizado el rendimiento de esta puntuación como herramienta de cribado para la detección de DM2 no diagnosticada y prediabetes muestran que los puntos de corte óptimos varían ampliamente, desde 9 hasta 15 ^11^^,^^16^^,^^18^^,^^21^^,^^30^^,^^45^^,^^47^^,^^48^.

La principal diferencia entre las escalas analizadas fue los distintos puntos de cortes para la CC ^10^^,^^27^^,^^30^^,^^36^. En la escala CUBDRISC los puntos de corte de la CC utilizados son más bajos ^36^^)^ que aportan 4 puntos y la FINDRISC le da valor de cero a los hombres cuando la CC fue < 94 cm y una puntuación de 3 a las mujeres con mediciones de 80 a 88 cm ^10^. En contraste en la escala LA-FINDRISC el punto de corte de la CC es mucho más alto para ambos sexos, por tanto, todos los valores por debajo de 94 cm para hombres y 90 cm para mujeres no aportan puntos (cero) ^27^^,^^29^.

Al analizar todos los casos (ambos sexos) el punto de corte óptimo fue ≥ 13 para identificar personas con disglucemia en los cuestionarios FINDRISC y CUBDRISC que fue similar a lo hallado por otros autores ^12^^,^^21^^,^^28^^,^^45^. En contraste, el punto de corte óptimo para el cuestionario LA-FINDRISC fue ≥ 11, el cual es más bajo que lo reportado por otros investigadores (≥ 14) ^30^ y cercano a lo obtenido por Bernabe-Ortiz *et al*. (≥ 10) ^28^^)^ y Nieto-Martínez *et al.* (≥ 9) ^(^^29^*.* El empleo de la escala CUBDRISC podría recomendarse para evaluar el riesgo de disglucemia en población cubana, teniendo en cuenta que utiliza los puntos de cortes de la circunferencia de la cintura para población cubana ^36^.

Consideramos que en esta investigación las puntuaciones óptimas obtenidas para las escalas FINDRISC, LA-FINDRISC y CUBDRISC para detectar personas con disglucemia y diabetes, aumentan la especificidad de la encuesta, disminuye así el número de casos falsos positivos, incrementa el índice de verosimilitud positivo y muestra también una mejor área bajo la curva ^16^^,^^31^. No obstante, para la identificación de sujetos con prediabetes obtuvimos un punto de corte optimo ≥ 12 puntos para las escalas FINDRISC y CUBDRISC, en cambio para la LA-FINDRISC fue ≥ 11 puntos. Con relación a los puntos de cortes óptimos para la identificación de DM2 en las escalas FINDRISC, LA-FINDRISC y CUBDRISC (13, 12 y 14, respectivamente) fueron un poco más altos que para la prediabetes. Los puntos de cortes al emplear el cuestionario FINDRISC en varios estudios mostraron resultados cercanos (puntuaciones de 11, 13, 14 y 15) para la detección de personas con DM2 ^16^^,^^21^^,^^25^^,^^28^^,^^45^.

Para los puntos de corte óptimos, la literatura presenta varias diferencias cuando se utiliza la PTGO para el diagnóstico de disglucemias, por ejemplo; Villena *et al*. ^45^ (FINDRISC, Perú) hacen referencia al valor ≥ 13 para detectar DM2. Gabriel *et al.* (FINDRISC, Europa) ^(^^11^, mencionan un punto de corte óptimo de ≥ 14 para identificar personas con disglucemia. Muñoz-González *et al.* (Venezuela) ^30^ encontraron que una puntuación ≥ 14 puntos predice la diabetes mellitus o trastornos del metabolismo de la glucosa y sugirieron que los pacientes con LA-FINDRISC ≥ 14 puntos deben realizarse una prueba de tolerancia a la glucosa oral ^30^. Makrilakis *et al.*^16^ en Grecia (FINDRISC) mencionan un valor ≥ a 15 como el punto óptimo para predecir DM2. Gomez-Arbelaez *et al.*^25^ en Colombia (FINDRISC), obtuvieron un punto de corte optimo ≥ 14 puntos para identificar DM2. Bernabe-Ortiz *et al.*^28^ en Perú (FINDRISC), mostraron como punto de corte optimo ≥ 11 para detectar casos de DM2. En España (FINDRISC), Salinero-Fort *et al.*^21^ hallaron que el mejor punto de corte fue ≥ 13, basado únicamente en los criterios de la PTGO.

En esta investigación para las distintas escalas (FINDRISC, LA-FINDRISC y CUBDRISC) se encontró valores de la AUC-ROC de 0,831, 0,833 y 0,831, respectivamente para personas con prediabetes, así como 0,877, 0,880 y 0,877, respectivamente para personas con DM2 (valores considerado de muy buena precisión), superior a los descritos por otros autores ^10^^-^^12^. Lo cual es lógico, ya que el cuestionario se aplicó a personas con al menos un factor de riesgo de diabetes.

Asimismo, al analizar la población de estudio de acuerdo con el sexo, encontramos el valor de corte óptimo ≥ 11 en hombres para predecir disglucemia tanto con la escala FINDRISC y LA-FINDRISC como para la CUBDRISC. Sin embargo, para las mujeres los valores óptimos para identificar personas con disglucemia fueron de ≥ 13 para el FINDRISC y de ≥ 14 para el CUBDRISC, resultados similares se han encontrado en otros estudios que utilizaron el FINDRISC ^25^^,^^47^^,^^49^. En el caso de la escala LA-FINDRISC para mujeres el valor óptimo para detectar disglucemia fue también ≥ 11 semejante al de otros estudios ^27^^,^^29^^,^^30^.

No recomendamos utilizar en Cuba la escala LA-FINDRISC debido a que las mediciones de la circunferencia de la cintura son mucho más altas ^26^^,^^29^ que las usadas en Cuba ^35^, además de reducir la puntuación al riesgo de desarrollar diabetes, así como detecta menos sujetos con GAA y TGA que la escala CUBDRISC.

Nuestros resultados sugieren que un valor de corte ≥ 11 en hombres y ≥ 12 en mujeres (punto de corte para identificar prediabetes) es esencial para indicar una PTGO en la búsqueda tanto de diabetes no diagnosticada como prediabetes ^22^^,^^48^. En este estudio con la aplicación de las escalas FINDRISC y CUBDRISC se demostró que las personas con puntaje ≥ 13 tienen mayor riesgo de padecer disglucemia. Distintos trabajos de validación de la escala FINDRISC, presentaron punto de cortes similares (entre 12 a 14) al de esta investigación para la detección de disglucemia ^11^^,^^12^^,^^25^.

El uso de las escalas de riesgo FINDRISC y CUBDRISC nos permite definir la población con mayor riesgo de DM y, en consecuencia, intervenir oportunamente para eliminar o retardar su aparición. Los esfuerzos en ese sentido incluyen la sensibilización de los médicos de atención primaria, así como el desarrollo de políticas públicas de prevención y educación pública desde edades tempranas ^26^.

El diseño transversal del estudio constituye una limitación; por lo tanto, nuestra recomendación es realizar un seguimiento de diez años o más de la cohorte para validar nuestras predicciones de riesgo cohorte para validar nuestras predicciones de riesgo ^35^. Otra de las limitaciones es que los participantes provienen de una pesquisa activa, por lo tanto, los resultados pueden no ser extrapolados al resto de la población cubana. La mayoría de las personas eran mujeres, y esta situación puede explicarse en parte porque hay mayor cumplimiento en la asistencia de las citas médicas, son más propensas a participar en los programas de promoción y prevención y en completar cuestionarios.

Una tercera limitación tiene que ver con el hecho que no se les realizó la PTGO a las personas que no presentaban factores de riesgo de diabetes, por lo que no se pudo determinar sensibilidad y especificidad de los cuestionarios utilizados, ni calcular las curvas ROC en esos individuos. Sin embargo, el tamizaje de diabetes en el presente estudio no era dirigido a la comunidad general, que enfatiza la aplicación de los cuestionarios FINDRISC; LA-FINDRISC y CUBDRISC en personas con factores de riesgo de diabetes.

Se puede afirmar, por tanto, que el cuestionario FINDRISC ha sido utilizado ampliamente, y que se ha probado en la mayoría de los casos, que su uso resulta beneficioso para la detección oportuna, aportando además el beneficio del bajo costo y facilidad en su aplicación ^10^^-^^12^^,^^21^^,^^27^^-^^29^^,^^43^. Por ello el cuestionario CUBDRISC puede ser considerado también una alternativa para el tamizaje para identificar personas con disglucemias en población cubana.

El empleo de las escalas de riesgo FINDRISC y CUBDRISC es un instrumento sencillo, rápido, no invasivo, fidedigno y económico que permitirá identificar individuos con riesgo de disglucemia, que pudiera incorporarse al programa de diabetes y de medicina familiar en Cuba, y serviría de referencia a otras poblaciones de la región con características étnicas similares a las nuestras. El uso de estas escalas no solo facilita la detección de personas con niveles altos de glucosa o diabetes no conocida, sino que también permite excluir a personas de la realización de una PTGO.

Se concluye que existe una concordancia casi perfecta entre las escalas. El puntaje CUBDRISC demostró ser una herramienta útil para identificar personas con disglucemia en una población cubana. Definió que las personas con puntaje ≥ 13 tienen mayor riesgo de padecer alguna disglucemia y aquellas con puntuación ≥ 12 y ≥ 14 poseen mayor riesgo de prediabetes y diabetes, respectivamente. Sin embargo, son necesarios otros estudios similares a este en otras regiones de Cuba.
